# Novel Therapeutic Hybrid Systems Using Hydrogels and Nanotechnology: A Focus on Nanoemulgels for the Treatment of Skin Diseases

**DOI:** 10.3390/gels10010045

**Published:** 2024-01-06

**Authors:** Kamil Sghier, Maja Mur, Francisco Veiga, Ana Cláudia Paiva-Santos, Patrícia C. Pires

**Affiliations:** 1 Faculty of Pharmacy, Masaryk University, Palackého tř. 1946, Brno-Královo Pole, 612 00 Brno, Czech Republic; 2 Faculty of Pharmacy, University of Ljubljana, Aškerčeva c. 7, 1000 Ljubljana, Slovenia; 3 Faculty of Pharmacy, University of Coimbra, Azinhaga de Santa Comba, 3000-548 Coimbra, Portugal; fveiga@ci.uc.pt; 4 REQUIMTE/LAQV, Group of Pharmaceutical Technology, Faculty of Pharmacy, University of Coimbra, 3000-548 Coimbra, Portugal; 5 CICS-UBI—Health Sciences Research Centre, University of Beira Interior, 6201-001 Covilhã, Portugal

**Keywords:** anti-aging, nanoemulgels, nanoemulsions, neuropathy, skin cancer, skin infection, skin inflammation, topical administration, transdermal administration, wound healing

## Abstract

Topical and transdermal drug delivery are advantageous administration routes, especially when treating diseases and conditions with a skin etiology. Nevertheless, conventional dosage forms often lead to low therapeutic efficacy, safety issues, and patient noncompliance. To tackle these issues, novel topical and transdermal platforms involving nanotechnology have been developed. This review focuses on the latest advances regarding the development of nanoemulgels for skin application, encapsulating a wide variety of molecules, including already marketed drugs (miconazole, ketoconazole, fusidic acid, imiquimod, meloxicam), repurposed marketed drugs (atorvastatin, omeprazole, leflunomide), natural-derived compounds (eucalyptol, naringenin, thymoquinone, curcumin, chrysin, brucine, capsaicin), and other synthetic molecules (ebselen, tocotrienols, retinyl palmitate), for wound healing, skin and skin appendage infections, skin inflammatory diseases, skin cancer, neuropathy, or anti-aging purposes. Developed formulations revealed adequate droplet size, PDI, viscosity, spreadability, pH, stability, drug release, and drug permeation and/or retention capacity, having more advantageous characteristics than current marketed formulations. In vitro and/or in vivo studies established the safety and efficacy of the developed formulations, confirming their therapeutic potential, and making them promising platforms for the replacement of current therapies, or as possible adjuvant treatments, which might someday effectively reach the market to help fight highly incident skin or systemic diseases and conditions.

## 1. Introduction

### 1.1. The Skin: Properties and Advantages and Limitations as a Drug Delivery Route

The skin consists of three primary layers: the epidermis, the dermis, and the hypodermis [1,2,3]. The epidermis, the outermost layer, is divided into several sublayers, with the deepest layer being known as the *stratum basale* (also called the basal layer). This layer contains rapidly dividing basal cells that continually undergo mitosis to replace the cells lost from the skin’s surface. As these basal cells divide and mature, they gradually move upwards toward the skin’s surface [4,5]. Above the *stratum basale* lies the *stratum spinosum*, which provides strength and support to the epidermis. The cells in this layer have spiny projections that interlock with neighboring cells, enhancing tissue integrity [6,7]. Further up is the *stratum granulosum*, where the cells begin to produce large amounts of keratin and other proteins. As these cells mature, they form flattened, granular layers, preparing to become the outermost protective barrier of the skin [7,8]. Finally, we have the *stratum corneum*, the outermost layer of the epidermis. This layer is composed of tough, flattened, and fully keratinized cells known as corneocytes. These corneocytes are continuously shed and replaced by new cells from the lower layers. The *stratum corneum* acts as a formidable barrier, preventing the entry of pathogens and chemicals, and excessive water loss [9,10,11]. Beneath the epidermis lies the dermis, a thicker and more complex layer of the skin, primarily composed of connective tissue, which includes collagen and elastic fibers, providing structural support and elasticity to the skin. The dermis houses blood vessels, nerves, hair follicles, sebaceous glands, and sweat glands. It also contains sensory receptors and specialized cells like Merkel cells, responsible for detecting touch and pressure [12,13,14]. Lastly, the hypodermis, or subcutaneous tissue, forms the deepest layer of the skin. It is mainly composed of adipose tissue (fat) that acts as an insulator and cushion, regulating body temperature and providing protection to underlying organs and structures [15,16].

Several factors can affect the properties of the skin. Environmental factors such as ultraviolet (UV) radiation from the sun can cause skin damage, premature aging, and increase the risk of skin cancer. Pollution, chemicals, and harsh weather conditions can also impact the skin’s health and appearance [17,18,19]. Additionally, lifestyle choices, such as diet, smoking, and alcohol consumption, can also influence the skin’s elasticity and overall health. Hormonal changes, stress, and certain medical conditions may also lead to skin issues, which can consequently evolve into pathological conditions, such as acne, eczema, and psoriasis, among others [20,21,22,23]. Understanding the intricate organization of the skin’s layers and their functions is essential for maintaining healthy skin. Proper care, protection from harmful environmental factors, and a balanced lifestyle can contribute to the overall health and well-being of this remarkable organ [24,25].

Furthermore, the fact that the skin, as the body’s largest external organ, serves as a protective barrier against various external factors, makes drug delivery on or through the skin a significant challenge, since the skin exhibits a very low or even nonexistent permeability to most drug molecules [26,27,28]. Factors such as *stratum corneum* composition, hydration, anatomical site, and individual variations contribute to the overall high complexity of skin drug permeation, and the understanding of the skin’s characteristics is essential for optimum drug delivery, in both topical and transdermal administration [24,29,30].

Transdermal and topical drug administration are both methods of delivering medications to the body through the skin. However, there are some key differences between the two [31,32]. Transdermal application refers to the delivery of medications through the skin, and into the bloodstream, allowing active ingredients to have systemic effects since they are meant to be distributed throughout the body. Hence, the formulation is designed to penetrate the skin’s surface and reach the bloodstream [33,34]. It is commonly used for medications that require slow, continuous release into the bloodstream over an extended period of time, and often used for systemic conditions such as hormone replacement therapy, pain management, or nicotine replacement therapy, among other applications [35,36,37]. Drugs are usually delivered through patches, gels, or creams, specifically designed to facilitate absorption through the skin and controlled release into the bloodstream [38,39,40,41]. On the other hand, topical administration involves applying medications directly to the skin’s surface to exert local effects on the area of application. In this case, the drug is meant to stay primarily at the site of application and will probably not be designed to penetrate deeply into the skin’s layers and reach the bloodstream [42,43,44]. Hence, this administration route is typically used for localized conditions, such as skin infections, rashes, inflammation, and other skin-related issues [45,46,47]. Formulations are designed to target specific areas without affecting the entire body, and come in various forms, including creams, ointments, lotions, sprays, and foams, depending on the intended application [48,49,50].

Nevertheless, although topical and transdermal drug delivery have gained significant interest due to their numerous advantages, including noninvasiveness, easy administration, and possibility of a localized therapy, conventional pharmaceutical forms often lack an answer to the many challenges that drug delivery on or through the skin presents, such as drug permeation and/or retention, drug metabolism by the skin’s enzymes, or effective solubilization of hydrophobic drugs [33,51,52,53]. Here, novel approaches using nanotechnology for drug encapsulation and delivery can be the answer.

### 1.2. Nanotechnology for Efficient Skin Drug Delivery: A Special Focus on Nanoemulsions and Nanoemulgels

**Figure 1:**
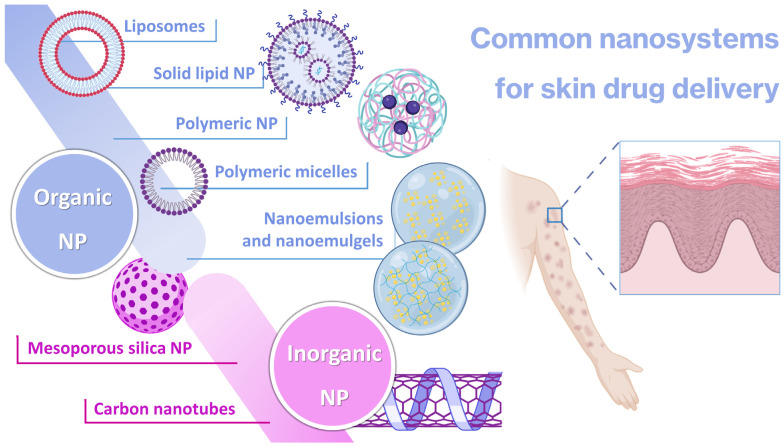
Schematic representation of common nanosystem categories for skin drug delivery (produced with Biorender).

Nanosystems are generally characterized as being structures in the nanosize range which are capable of encapsulating drug molecules for improved drug delivery. Several different types of nanosystems have been developed over the years, each with their own specific composition and characteristics (Figure 1) [54,55,56,57].

Nanoparticles (NPs) are the most common general type of nanosystems, with the term typically referring to particles within the size range of 1 to 100 nm [58,59]. Their adjustable physicochemical properties, such as size, shape, and surface characteristics, contribute to tailored and improved physiological performance, when compared to conventional pharmaceutical forms, generally leading to more effective treatment and minimization of the side effects [60,61,62,63]. NPs also hold significant potential for targeted delivery of active ingredients to specific locations, in contrast to conventional formulations which usually require the administration of larger quantities of active ingredients in order to reach a therapeutically effective response, and often fail to target the desired area of interest, resulting in an extensive and potentially harmful penetration of the drugs into healthy tissues, hence causing systemic side effects [60,64,65]. Key considerations include these particles’ size and structure, which is essential to optimize their performance and minimize potential harm, with surface modification by different types of substances, such as polymers and other molecules, being important in what concerns stability, specificity, and therapeutic effectiveness [66,67,68]. NPs can be divided into various subcategories, including organic and inorganic nanocarriers [69,70,71].

Inorganic NPs include mesoporous silica nanoparticles (MSNs), which are nanoscale structures composed of silica, with large surface areas and well-defined pores. Their ordered porous structure, with uniform pore sizes ranging from 2 to 50 nm, allows for efficient encapsulation and controlled release of active substances [72,73]. The large surface area of MSNs provides a space for active ingredient loading, leading to high drug-loading capacity and enhanced therapeutic efficacy, but despite these advantages, MSN synthesis requires precise control over particle size, pore site, and surface chemistry, which can be complex and time-consuming [74,75,76]. Carbon nanotubes (CNTs) are another type of inorganic nanocarrier, being cylindrical structures composed of carbon atoms arranged in hexagonal lattice, producing a large surface area that offers significant loading capacity for efficient encapsulation of therapeutic agents [77,78]. They possess unique advantages, such as high mechanical strength, exceptional electrical conductivity, thermal stability, protection of incorporated substances from degradation, controlled release capacity, and targeting ability [79,80,81]. These properties, combined with their nanoscale dimensions, make CNTs attractive candidates for therapeutic applications. Nevertheless, they have quite significant disadvantages, especially their poor water solubility and potential for toxicity [82,83,84]. Given the potential toxicity of inorganic nanoparticles, among other disadvantages, organic nanocarriers have been preferred for various applications [85,86].

There are many types of organic nanocarriers, all having in common high biocompatibility with the human body, an essential criterion for drug delivery in order to minimize toxicity and side effects [87,88]. Liposomes, the first to be developed, and also the first to effectively reach the pharmaceutical market, are spherical nanocarriers with single (small or large unilamellar vesicles) or multiple (multilamellar vesicles) bilayered membranes, formed of natural or synthetic lipids, which enclose an aqueous core [89,90,91,92]. They have showed a number of advantages compared to conventional systems, including enhanced drug delivery, drug protection from degradation, improved bioavailability, and even prolonged half-life in the blood circulation when functionalized with specific polymers (such as polyethylene glycol (PEG)) on their surface (stealth or PEGylated liposomes) [82,93,94]. On the other hand, solid lipid nanoparticles (SLNs) are formed by dispersing melted solid lipids in water, while emulsifiers are employed to stabilize the dispersion. These nanocarriers provide a lipophilic lipid matrix that facilitates the dispersion or dissolution of lipophilic drugs, providing controlled drug delivery, biocompatibility, high drug payload, and improved bioavailability of poorly water-soluble drugs [82,95,96]. Nevertheless, some important disadvantages of SLNs reside in their inability to encapsulate hydrophilic drug molecules, and stability issues have been reported, including burst release of the drugs during storage [97,98]. Another relevant nanocarrier subcategory is the polymeric micelles (PMs), self-assembled nanostructures formed by amphiphilic block copolymers in aqueous solutions. These nanosystems consist of a hydrophobic core, inside which hydrophobic drugs can be encapsulated, and a hydrophilic shell, stabilizing the micelle, which is made possible by the unique structure of block copolymers. These structures can also be surface-functionalized with targeting ligands to achieve specific delivery to target cells or tissues [99,100,101]. On the other hand, also composed of polymers, polymeric nanoparticles (PNPs) are nanocarriers that are fabricated using biocompatible polymers, typically poly (lactic-co-glycolic acid) (PLGA), PEG, chitosan, or polycaprolactone (PCL), amongst others. PNPs have the ability to encapsulate and protect various types of drugs including small molecules, proteins, peptides, and nucleic acids. The encapsulation also improves their stability, and controls their release kinetics, which allows for sustained or targeted drug delivery [102,103,104]. However, both PMs and PNPs have been reported to exhibit toxicity, slow clearance, formulation instability issues due to aggregation, and low drug-loading capacity [102,105,106].

Additionally, all mentioned nanocarrier types have complex preparation methods, which are time-consuming, costly, and often lead to scale-up issues, while also many times not being environment-friendly due to the use of organic solvents and the need for high energy amounts during production [107,108,109].

Given the overall stability, toxicity, low drug-loading, and problematic preparation issues that are seen with other nanocarrier types, nanoemulsions and nanoemulgels have arisen as advantageous solutions with high potential. Nanoemulsions are kinetically stable biphasic dispersions of two immiscible phases, an oil phase and a water phase, which are combined with surfactants and/or cosurfactants to increase their stability. Two main types of nanoemulsions exist, water-in-oil (W/O) or oil-in-water (O/W), with the latter being the most common [110,111,112]. Usually, O/W nanoemulsions contain from 5% to 20% oils/lipids, with this amount increasing up to 70% when the nanoemulsion is W/O. The type of the oil used in the formulation of nanoemulsions depends on the active substances that are intended to be solubilized, with the selection often being performed based on a solubility criterion (oils that solubilize the active substance the best) [111,113]. On the other hand, surfactants are amphiphilic molecules, which are used to stabilize the nanoemulsions, to reduce the interfacial tension and prevent the aggregation of the internal phase droplets. They usually have an ability to absorb quickly at the oil–water interface, and provide steric, electrostatic, or dual electro-steric stabilization. Cosurfactants may be used as surfactant complements to strengthen the interfacial film, if they fit suitably in areas which are structurally weaker [114,115,116]. There is an ongoing effort in both industrial preparations and scientific works to ensure that the components which are used in the development of nanoemulsions be strictly nontoxic, generally-regarded-as-safe (GRAS) excipients, in order to ensure maximum biocompatibility and decrease the propensity for side effects potentiated by the formulation [117,118]. Nanoemulsions can be produced by high-energy, low-energy, or low- and high-energy combination methods [117,119]. High-energy emulsification methods depend on mechanical devices, which use energy for creating powerful disruptive forces to reduce the size of the formed droplets. Ultrasonicators, microfluidizers, and high-pressure homogenizers can be used, including on an industrial level. These methods have the advantage of being able to nanoemulsify almost any oil, but the dependence on instrumental techniques is associated with high costs and the generation of high temperatures, which might not be feasible for all formulation components (for example, thermolabile drugs) [120,121]. Hence, low-energy methods, such as phase inversion or spontaneous emulsification, are the most advantageous, since it is the energy stored in the system that is used to produce the ultrafine droplets, leading to lower production costs and an easy application [122,123]. One of the most used low-energy methods is the phase inversion temperature method, to take advantage of changes in the aqueous/oil solubility of surfactants in response to temperature fluctuation. This will include the conversion of a W/O to an O/W nanoemulsion, or the reverse, via an intermediary bicontinuous phase. The change of temperature from low to high causes the opening and reversal of interfacial structure, which leads to phase inversion. However, this method has a substantial disadvantage, which is that it cannot be used for thermosensitive drugs [124,125,126]. Hence, the most beneficial low-energy method ends up being spontaneous emulsification, in which the components are usually just mixed with each another, resorting to manual mixing or low-energy mixers (such as mechanical stirring or vortex stirring). This, of course, only happens when the right components are mixed in the right proportions, with the oils, surfactants and cosurfactants having to be miscible with one another, and with reasonably high amounts of surfactants/cosurfactants being valuable for maximum formulation stabilization, homogeneity, and low droplet size [127,128,129]. Regardless of the used preparation method, nanoemulsions’ droplet size usually varies between 20 and 500 nm, hence being small and responsible for these formulations’ clear or hazy appearance [117,130]. This type of nanosystem can be used in many different dosage forms, such as creams, sprays, gels, aerosols, or foams, and various administration routes, such as intravenous, oral, intranasal, pulmonary, ocular, topical, or transdermal drug delivery [111,131,132]. They have several relevant advantages, such as a high solubilization capacity, great long-term physical stability, which reduces the propensity for conventional destabilization mechanisms to occur (such as creaming, coalescence, or Ostwald ripening), and a very large surface area available for drug absorption to occur [133,134,135]. Nevertheless, for certain applications, nanoemulsions might not be the ideal dosage form, since they tend to be fluid and do not usually have bioadhesion capability, which can lead to a short retention time of the preparation at the site of administration, an important parameter in transdermal and topical administration. In order to tackle these issues, nanoemulsions can be transformed into nanoemulgels [136,137].

Nanoemulgels are hybrid colloidal systems composed of nanosized oil droplets dispersed in an aqueous gel matrix, combining the properties of both nanoemulsions and hydrogels, which solve important issues such as spreadability and skin retention of the preparations by increasing their viscosity [136,138]. The methodology for preparing a nanoemulgel entails the creation of a gel-based formulation using common and already described emulsification techniques, similar to those that are used in the preparation of nanoemulsions [139,140]. As topical or transdermal administration systems, they function as drug reservoirs, facilitating the controlled release of the drug from the inner phase to the outer phase, and subsequently onto the skin. Aside from controlled drug delivery, nanoemulgels exhibit several advantages over alternative topical formulations, which include compatibility with the skin, high viscosity, adhesiveness, good spreadability, and long-lasting therapeutic effects [139,140]. In what concerns common excipients that are used as part of a nanoemulgel’s composition, oil selection is usually dependent on the intended hydrophobicity, viscosity, permeability, and stability in the formulated nanoemulsion, but oils from natural sources, such as oleic acid, are commonly used, or chemically modified oils with medium chain mono-, di-, or tri-glycerides, such as Capryol^®^ 90, Miglyol^®^ 812, or Labrafac™ [141,142,143]. Additionally, there are several categories of surfactants, and among the most used are cationic surfactants (such as amines and quaternary ammonium compounds, or lecithin), anionic surfactants (such as sodium bis-2-ethylhexylsulfosuccinate, or sodium dodecyl sulfate), zwitterionic surfactants (such as phospholipids), and nonionic surfactants (such as Tweens, Lauroglycol^®^ 90, Cremophor^®^ EL, or Cremophor^®^ RH 40) [137,144,145]. Frequently used cosurfactants include propylene glycol, PEG 400, ethyl alcohol, or Transcutol^®^, with alcohol-based cosurfactants hence being the most preferred due to their capability to split between oil and water phases, thereby improving their miscibility [139,146,147]. Lastly, there are different types of gelling agents used in nanoemulgel formation, including natural gelling agents such as bio-polysaccharides (such as pectin, carrageenan, alginic acid, locust bean gum, or gelatine), derivates of bio-polysaccharides (such as xanthan gum, starch, dextran, or acacia gum), as well as semisynthetic (such as hydroxypropyl cellulose, ethyl cellulose, and sodium alginate) and synthetic polymers (such as carbomers or poloxamers) [136,137,148].

**Figure 2:**
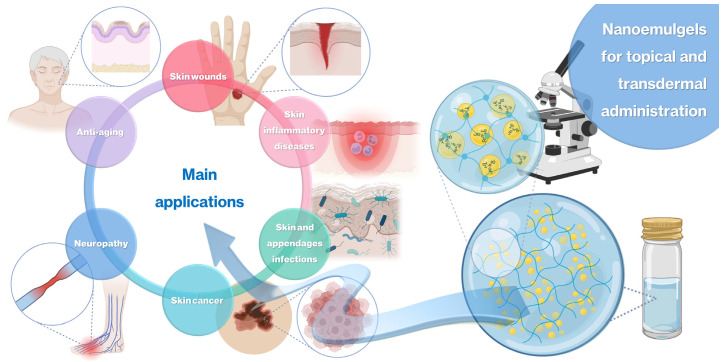
Schematic representation of nanoemulgel structure, and main applications of nanoemulgels in highly incident diseases, for topical and transdermal administration (produced with Biorender).

When developing a novel nanoemulgel, several properties have to be evaluated, in order to assess whether the preparation has optimum characteristics for the intended application, including droplet size, polydispersity index (PDI), zeta potential, pH, rheological properties, stability, bioadhesion, spreadability, in vitro drug release, ex vivo drug permeation, toxicity potential, and in vitro and/or in vivo assessment of the therapeutical potential of the developed formulation for the intended purpose. This review focuses on the latest advances regarding the development of novel nanoemulgels for transdermal or topical administration, for the treatment of several highly impactful diseases and conditions (Figure 2), such as skin wound healing, skin and appendage infections, skin inflammatory diseases, skin cancer, neuropathy, and skin aging. A critical analysis is performed regarding formulation composition and preparation, and all relevant characterization parameters, in order to assess the true potential of these formulations as novel functional platforms for drug delivery onto or through the skin (summary in Table 1).

## 2. Topical and Transdermal Nanoemulgels for the Treatment of Skin Diseases and Other Applications

### 2.1. Skin Wound Healing

**Figure 3:**
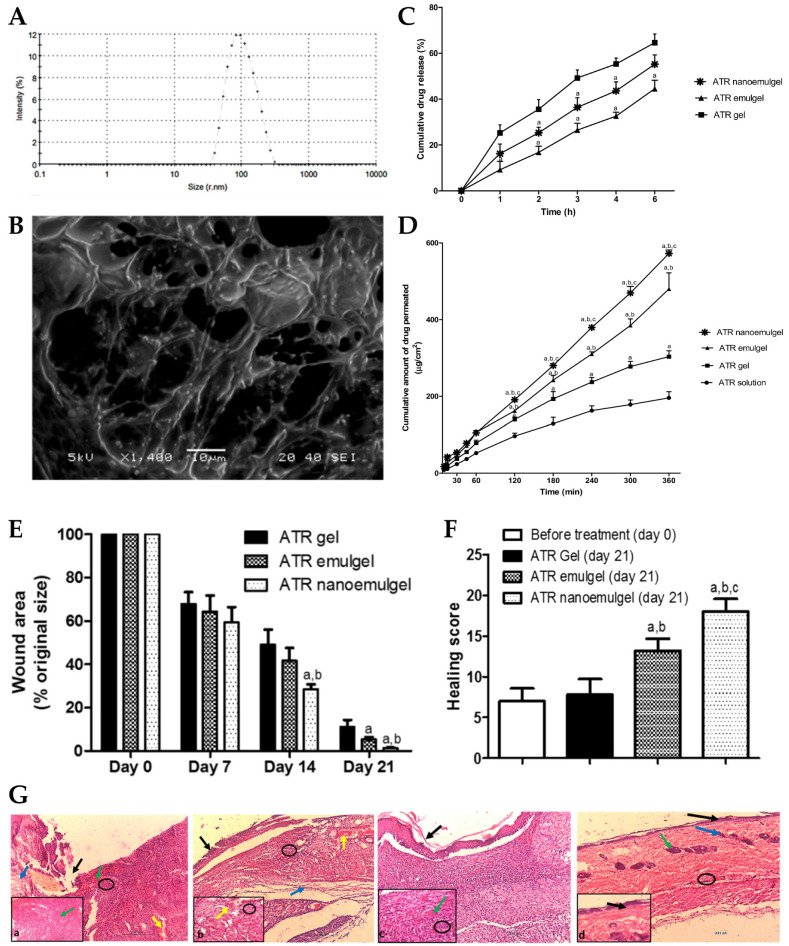
(**A**) Droplet size distribution of the developed ATR nanoemulgel; (**B**) surface morphology of the developed ATR nanoemulgel; (**C**) in vitro drug release profiles of the developed ATR nanoemulgel, compared to an emulgel and gel; (**D**) ex vivo drug permeation profiles of the developed ATR nanoemulgel, compared to an emulgel, a gel, and a solution; (**E**) wound area variation of rat skin after topical administration of the developed ATR formulations, after 0, 7, 14, and 21 treatment days; (**F**,**G**)—healing score (**F**) and photomicrographs (**G**) of rat skin before treatment (a), and after 21 days of topical administration of an ATR gel (b), an ATR emulgel (c), or an ATR nanoemulgel (d), where black arrows represent the absence of epidermal layer epithelization, blue arrows represent loss of collagen fibers normal arrangement in the dermal layer, green arrows represent severe congestion, yellow arrows represent hemorrhage, and black circles represent inflammatory cell infiltrations; ATR—atorvastatin; adapted from Morsy et al. [149], reproduced with permission from MDPI (Creative Commons CC BY 4.0 470 license).

Skin wounds are physical injuries of the skin tissue, which lead it to break and open. Wound healing is a complex process, which requires a coordinated series of cellular and molecular events, which aim to restore the integrity and functionality of damaged tissues [164,165,166]. It includes inflammation, cell migration, tissue formation, and remodeling, and due to lack of effectiveness or slow therapeutic action, new strategies are needed for efficient and fast wound healing [167,168,169]. In this context, Morsy et al. [149] developed a novel nanoemulgel formulation containing atorvastatin for wound healing application. Despite the fact that atorvastatin (ATR) is primarily prescribed as a lipid-lowering medication, to manage cholesterol levels, high cholesterol levels have been associated with impaired wound healing, which means that atorvastatin may indirectly contribute to improved wound healing outcomes [170,171,172]. Furthermore, this drug also shows anti-inflammatory properties, and angiogenic effects, which promote the formation of new blood vessels, leading to an adequate blood supply that is crucial for tissue regeneration [173,174,175]. Additionally, it has also been described as targeting and inhibiting the growth of microorganisms, including common wound pathogens, and has been linked to other pleiotropic effects, such as antioxidant properties, modulation of cellular signaling pathways, and promotion of cell proliferation and migration, which make this drug a quite relevant candidate due to multiple wound-healing-related beneficial effects [173,176,177]. The incorporation of ATR into a nanoemulgel formulation intended to allow a controlled and sustained release at the wound site, maximizes its potential therapeutic effects. Hence, the developed nanoemulgel was prepared by using a combination of high-pressure homogenization and ultrasonication techniques. First, the gel was prepared by adding sodium carboxymethyl cellulose (CMC) to water and stirring continuously until gel formation. After that, a primary O/W emulsion was made, containing ATR solubilized in a mixture of liquid paraffin, Tween^®^ 80, and propylene glycol, to which water was slowly added, and vortexed. Then, to this drug-loaded emulsion, the polymeric gel base was added and mixed for 5 min. Afterward, this primary emulgel was sonicated, for 10 min, in order to reduce its droplet size and finally obtain the required nanoemulgel. The developed formulations were characterized for particle size, PDI, zeta potential, viscosity, spreadability, in vitro drug release, stability, ex vivo permeation, and in vivo wound healing properties. The physicochemical characterization showed a small particle size of approximately 100 to 200 nm (Figure 3A,B), a good homogeneity with a PDI value of less than 0.3, and the zeta potential was found to be within the range of −20 to −30 mV, indicating good stability and preventing particle aggregation. Additionally, the viscosity and spreadability of the nanoemulgel was determined to be in the range suitable for topical application, ensuring ease of spreading and adherence to the wound area. The in vitro drug release profile (Figure 3C), determined across semipermeable cellulose membranes for 6 h, demonstrated a sustained release of the drug from the developed nanoemulgel over time, with a reduced initial burst release when compared to a CMC gel, and with a higher overall release when compared to an emulgel. Moreover, stability studies, which it underwent for a duration of 6 months, under storage conditions of 60% relative humidity and a temperature of 4 °C, indicated no noticeable alterations in several evaluated properties, such as color, appearance, spreading, or viscosity. In addition, an ex vivo permeation study (Figure 3D), through excised rat skin, revealed that the ATR nanoemulgel had a higher permeation, both in what concerns cumulative amount and velocity, than the drug-loaded emulgel, gel, or solution, after 2 h, also exhibiting the shortest lag time. Furthermore, a histopathological analysis of rats’ skin, after nanoemulgel topical application in an in vivo study, supported its positive effect on wound healing, showing reduced inflammation and increased angiogenesis, with a marked improvement in the skin histological architecture, and considerable healing after 21 days of ATR nanoemulgel treatment (Figure 3E–G). Additionally, although the developed gel-based formulations may encounter drawbacks such as limited residence time at the application site, the nanoemulgel formulation can address this concern by exhibiting enhanced retention in the affected area, and therefore prolonged retention, enabling a steady release of the drug, and facilitating extended contact with the skin surface.

**Figure 4:**
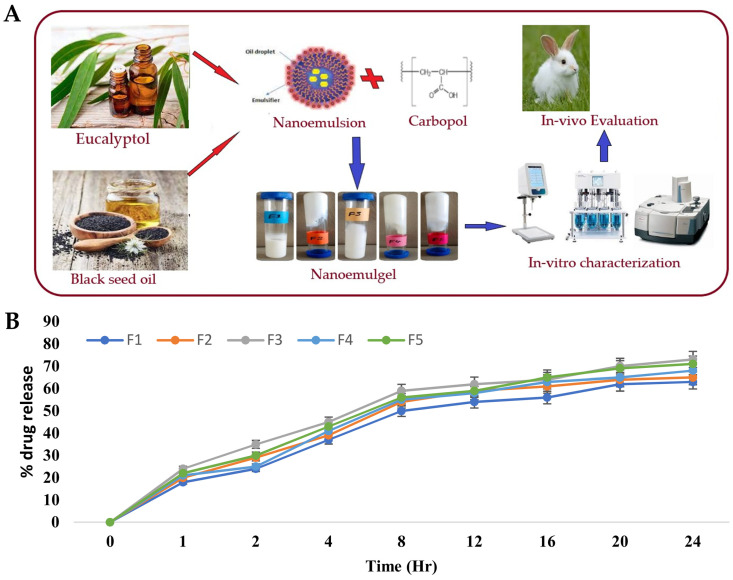
(**A**) Schematic representation of the developed eucalyptus oil nanoemulgel, including partial composition and general indication of performed studies; (**B**) in vitro eucalyptol release profiles of different nanoemulgels; adapted from Rehman et al. [150], reproduced with permission from MDPI (Creative Commons CC BY 4.0 470 license).

Wounds are strongly connected to health disorders such as immune system diseases, diabetes, chronic peripheral vascular disorders, and various infectious and inflammatory diseases. In this context, chronic wounds pose a significant healthcare challenge due to their slow healing and susceptibility to infections [178,179]. Eucalyptol has been reported to function as a good penetrant in transdermal and topical drug delivery systems and is also claimed to possess antibacterial properties against human and food-borne pathogens [180,181]. Hence, Rehman et al. [150] developed a nanoemulgel for wound healing incorporating eucalyptus oil, obtained from *Eucalyptus globulus*, into a nanoemulgel (Figure 4A), developing an effective platform designed to enhance the stability, permeation, and controlled release of eucalyptol, one of the main constituents of eucalyptus oil, thereby promoting its therapeutic efficacy in wound healing. The preparation of the nanoemulgel was divided into two steps, with the first including the preparation of different primary O/W nanoemulsions by solvent emulsification diffusion method. The nanoemulsions were made of an aqueous phase containing the hydrophilic surfactant Tween^®^ 80 and distilled water, and an oil phase containing black seed oil, the hydrophobic surfactant Span^®^ 60, and the cosurfactant/cosolvent propylene glycol. These two phases were mixed together using a magnetic stirrer, and then the nanoemulsion was produced by droplet size reduction using a high-speed homogenizer. From the selected primary nanoemulsion, nanoemulgels were created, where Carbopol^®^ 940 was used as the gelling agent. A Carbopol gel was produced by adding it to distilled water and mixing until a clear solution was formed, and then the pre-prepared nanoemulsion was added to the gel, in ratio of 1:1, with the pH being adjusted to a value of 5–6 using triethanolamine. These nanoemulgels were subsequently subjected to characterization. For the stability studies, temperature tests and centrifugation were used, with all formulations being subjected to storage at different conditions, namely, 8 °C, 25 °C, 40 °C, and 40 °C, with 40% relative humidity, for 28 days. Results showed that all the formulations were stable under the studied conditions, with no phase separation being observed after subsequent centrifugation. A Fourier-transform infrared spectrophotometer analysis was also employed to investigate the chemical interactions and compatibility between the components. By analyzing the produced spectra, it was possible to identify specific functional groups and molecular vibrations, confirming the absence of any major chemical changes or incompatibilities that could potentially affect the stability or therapeutic properties of the formulations. It was also shown that the pH had a major effect on the stability of the systems, as triethanolamine, used to adjust the formulation’s pH to simulate the pH of the skin, affected the transparency and disrupted the internal structure of the formulations. Furthermore, organoleptic homogeneity tests were performed, where changes in color, phase separation, consistency, and liquefaction were observed. All the formulations were observed to be off-white in color, and smooth in terms of consistency, and showed also reasonable to good spreadability. Additionally, the drug content analysis showed that the drug was uniformly distributed throughout the nanoemulgels. In the study of in vitro drug release (Figure 4B), the nanoemulgel which released the highest amount of drug was selected. The selected nanoemulgel’s particle size, PDI, and zeta potential were also determined. The droplet size was found to be around 139 ± 5.8 nm, the PDI was less than 0.45, and the zeta potential was measured to be −28.05 mV. After formulation physicochemical characterization, an in vivo study evaluated the wound-healing activity of the nanoemulgel in rabbits. The percentage of wound contraction was measured over a 15-day period, and they compared a negative control group, a nanoemulgel group, and a standard commercial product group. The results showed that the percentage of wound contraction for the nanoemulgel group on day 15 was 98.17%, indicating a significant improvement in wound healing compared to the negative control group (70.84%). Additionally, a statistical analysis using one-way ANOVA confirmed that the developed nanoemulgel had a wound-healing activity similar to that of the commercial cream, confirming its effectiveness in promoting wound healing. Thus, the performed comprehensive characterization studies, including physicochemical analysis and in vivo wound evaluations, provide valuable insights into the developed formulation’s potential efficacy. Overall, based on the provided information, we can conclude that the topically applied nanoemulgel formulation containing eucalyptus oil as an active ingredient demonstrated significant wound-healing activity and stability in the tested conditions, hence being a potentially novel and effective strategy for skin wound healing.

Diabetes mellitus remains a significant global health concern, affecting millions of individuals worldwide. One of the most debilitating complications of diabetes is the development of chronic, nonhealing wounds. These wounds pose substantial challenges for patients and healthcare providers, leading to increased morbidity, including the risk of limb amputation, and significant healthcare costs [182,183,184]. To address this critical issue, researchers are continuously exploring innovative approaches to improve wound healing and management. Hence, a study conducted by Yeo et al. [151] also focused on the fabrication and characterization of a topical nanoemulgel, containing tocotrienols and naringenin, for the management of diabetic wounds. In that study, researchers aimed to develop a nanoemulgel formulation taking advantage of the therapeutic potential of two bioactive compounds: tocotrienols, which are members of the vitamin E family with potent antioxidant and anti-inflammatory properties, and naringenin, a flavonoid known for its wound healing and tissue regeneration capabilities [185,186,187,188]. The study aimed to explore the synergistic effects of these compounds, with the goal of creating a multifunctional nanoemulgel that could accelerate wound closure, improve tissue repair, and alleviate the underlying inflammation often associated with diabetic wounds. With the aim of entrapping naringenin within the oil droplets of the o/w nanoemulgel, the oil phase was chosen according to the highest achievable solubilization for this compound. Among the tested oils, Capryol^®^ 90 demonstrated the highest solubilization of naringenin, and hence was selected. Furthermore, the addition of tocotrienols to Capryol^®^ 90 did not significantly impact the solubility of naringenin, suggesting successful and stable encapsulation of the drug with the chosen oil base. Additionally, in order to ensure the safety and biocompatibility of the formulation, the selection of appropriate surfactants is vital. Nonionic surfactants were prioritized due to their GRAS status and their ability to withstand pH or ionic strength changes. Solutol^®^ HS15, with an HLB value of 15.2, was chosen as the primary surfactant. It offers several advantages, including low toxicity, good biocompatibility, and permeation-enhancement ability, making it a suitable choice for this formulation. To further stabilize the nanoemulsion and achieve a flexible interfacial film, Transcutol^®^ P, a cosurfactant and cosolvent, was selected to work in combination with Solutol^®^ HS15. Transcutol^®^ P has been extensively studied for its skin-permeation-enhancing properties, without significantly affecting the diffusion of permeants across the skin. Moreover, it has the capability of creating an intracutaneous depot, increasing the reservoir capacity in the *stratum corneum* for incorporated therapeutics. These properties make Transcutol^®^ P an ideal choice for the development of a nanoemulgel, especially for topical application. The preliminary nanoemulsions were prepared using the spontaneous emulsification method, followed by sonication and vortexing for increased homogenization. For the drug-loaded nanoemulsions, naringenin was solubilized in the mixture of Capryol 90 and tocotrienols, and then the surfactant and cosurfactant were added. Distilled water was added dropwise with continuous stirring, to achieve a final naringenin concentration of 2 mg/mL. To formulate and optimize a stable primary nanoemulsion, several critical characteristics were considered, including droplet size, PDI, and zeta potential, as they significantly impact the in vivo stability and overall performance of the formulations. The mean droplet size for most blank nanoemulsions was found to be less than 200 nm, with a trend for increased droplet size with higher oil concentrations, which aligns with previous findings in the literature, where an increment in oil content usually leads to larger droplet sizes due to a reduction in specific surface area. The zeta potential of the preliminary nanoemulsions ranged from −4 mV to +11 mV. Although nanoemulsions with low absolute zeta potential values can theoretically be more prone to being unstable, due to low electrostatic repulsion, several studies have shown that stable nanoemulsions can be formulated using nonionic surfactants with low surface charge values. This observation was confirmed in the study, where the formulated nanoemulsions, containing nonionic surfactants, exhibited good thermodynamic stability, in thermodynamic stability studies involving centrifugation, heating–cooling cycles, and freeze–thaw cycles, with no signs of phase separation, cracking, or creaming, hence ensuring their potential as stable delivery systems. The stable and optimized naringenin nanoemulsion was chosen as the starting point to formulate the nanoemulgels, being mixed with 1%, 1.5%, or 2% *w*/*v* Carbopol^®^ 934 or Carbopol^®^ 940 gel bases. The ratio of naringenin nanoemulsion to gel base was 1:1, and the mixture was stirred for 10 min at 500 rpm. Triethanolamine was used to adjust the pH to 4.9–5.3, ensuring a slightly acidic environment beneficial for wound healing. The nanoemulgels were allowed to stand for 24 h to remove trapped air. The addition of the gelling agent led to a significant reduction in the size of the dispersed droplets. Furthermore, the zeta potential of the nanoemulgels decreased when compared to the optimized nanoemulsion, indicating a more negative surface charge, attributed to the presence of carboxylate ions on the Carbopol molecules. The incorporation of the gel matrix also further stabilized the dispersed oil droplets, indirectly supporting the stability of the nanoemulgel formulations. The optimized nanoemulgel had nanometric globules (145.6 ± 12.5 nm) with a PDI of 0.452 ± 0.03 and a zeta potential of −21.1 ± 3.32 mV. The spreadability of the formulations decreased with an increase in gelling agent concentration, which is typical for fluid gels that are easily spread over the affected area. The optimized nanoemulgel showed good spreadability and a high viscosity of 297600 cP. The nanoemulgels also showed promising mucoadhesive properties, with better adhesion being proportional to the concentration and grade of Carbopol that was used. In vitro drug release studies, in phosphate buffer saline (PBS), showed that Carbopol^®^ 934-containing nanoemulgels exhibited higher drug release compared to those with Carbopol^®^ 940, possibly due to their lower viscosity. Additionally, an initial burst release was observed in the first 2 h of the assay, which has been deemed typical of polymer-matrix-based formulations. The in vitro drug release of naringenin from the nanoemulgel was revealed to have controlled and sustained characteristics, adding up to 74.62 ± 4.54% within a time period of 24 h. Hence, overall, the findings from that study provide valuable insights into the formulation of stable naringenin nanoemulsions and nanoemulgels, paving the way for potential applications in wound healing treatments and other topical delivery approaches. Further studies and clinical evaluations may be needed to fully explore the therapeutic potential of the developed formulations in wound healing or other dermatological conditions.

**Figure 5:**
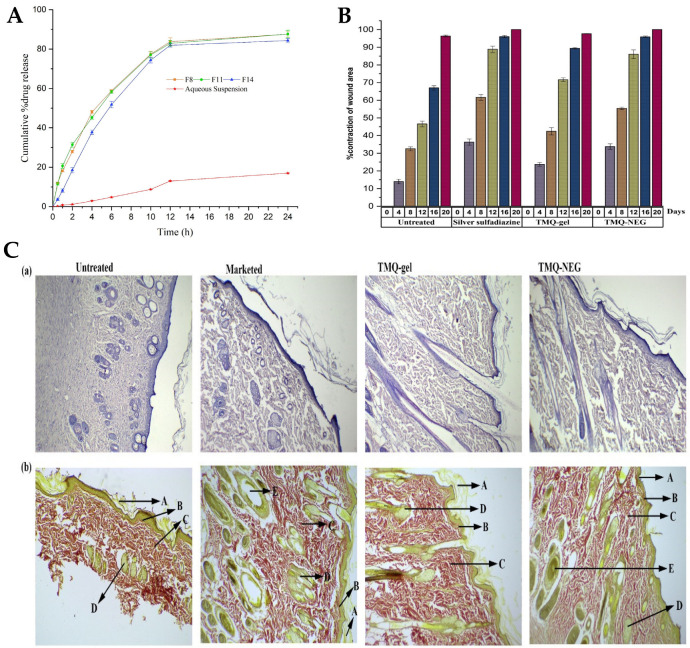
(**A**) In vitro drug release profiles of the TMQ-loaded nanoemulsions, compared to a drug aqueous suspension; (**B**) percentage of contraction of wound area variation, in the in vivo study, assessed for 20 days, with topical application of the developed TMQ nanoemulgel (TMQ-NEG), a conventional TMQ gel (TMQ-gel), a silver sulfadiazine formulation (Silver sulfadiazine), or no treatment (Untreated); (**C**) histopathology analysis of the rat’s skin at day 20, newly healed, after topical application of the developed TMQ nanoemulgel (TMQ-NEG), a conventional TMQ gel (TMQ-gel), a silver sulfadiazine formulation (Marketed), or no treatment (Untreated), stained with hematoxylin-eosin (a) or Van Gieson (b), with arrows indicating the stratum corneum (A), the papillary dermis (B), collagen fibers (C), sebaceous glands (D), or hair follicles (E); adapted from Algahtani et al. [152], reproduced with permission from MDPI (Creative Commons CC BY 4.0 470 license).

Algahtani et al. [152] also aimed to develop a novel topical nanoemulgel formulation, loaded with thymoquinone (TMQ), and evaluate its effectiveness in wound healing. TMQ is a bioactive compound derived from *Nigella sativa*, and it has been widely studied for its potential therapeutic effects, including antioxidant, antimicrobial, anti-inflammatory, and wound healing properties. It has shown promise in promoting wound closure and tissue regeneration. However, its effectiveness in wound healing is limited by poor water solubility and low skin permeation [189,190,191]. Hence, the study aimed to develop a nanosystem formulation, encapsulating TMQ within the internal phase’s oil droplets, using black seed oil as a natural carrier, and stabilized by a surfactant and cosurfactant mixture, with the addition of the aqueous phase leading to a primary O/W nanoemulsion. To prepare the TMQ-loaded nanoemulsion, a high-energy method was used, employing an ultrasonication technique. Initially, the researchers prepared a coarse emulsion by combining 5% *w*/*w* (50 mg/g) of TQM with the mixture of the oil phase and a surfactant/cosurfactant mixture (Kolliphor^®^ EL/Transcutol^®^ HP). The components were mixed by using a vortex mixer, and then added to the aqueous phase, while continuously vortexing for 1 min. Then, in order to improve the emulsion’s properties, the coarse emulsion was subjected to ultrasonication, using an ultrasonic homogenizer in a water bath. This process helped to break down larger droplets into smaller ones, and enhance the stability of emulsion, transforming it into a nanoemulsion. The increase in surfactant mixture concentration decreased the mean droplet size, and the ultrasonication time also significantly influenced the mean droplet size and PDI of the nanoemulsions. When the ultrasonication time increased from 3 to 5 min, the mean droplet size decreased. Nevertheless, an ultrasonication time higher than that, leading to an excessive exposure to ultrasonication energy known as overprocessing, caused intense turbulence, leading to collisions between the nanoemulsion’s droplets and their subsequent coalescence, resulting in larger droplet sizes. The average globule sizes of selected nanoemulsions varied between 40.02 and 99.66 nm, and the PDI value between 0.052 and 0.542. Nanoemulsion viscosity was also measured, being between 71.04 mPas and 88.82 mPas, and drug content varied between 98.74% and 99.32%. The zeta potential of these primary nanoemulsions was measured to be in the range of −26.7 to −30.6 mV, which is expected to contribute to their stability, since high repulsive forces between the nanoemulsion droplets might help prevent their coalescence. Furthermore, the preliminary formulations’ stability was effectively assessed, and all were found to be stable when subjected to various tests, including heating–cooling cycles, centrifugation, and freeze–thaw cycles. The formulations that demonstrated thermodynamic stability were selected for the in vitro drug release studies (Figure 5A) using the dialysis bag technique. The bags were filled with 1 mL of TMQ nanoemulsion formulation, and throughout the study, at specific time intervals, aliquots were withdrawn and replaced with PBS, up to 24 h. The aliquots were then analyzed by using UV-spectroscopy to quantify the amount of TMQ released from each formulation. After 12 h, more than 80% of the drug was released from all screened nanoemulsions, with the maximum cumulative drug release (at 24 h) varying between 84.3% and 87.1%. Additionally, comparing the TMQ nanoemulsions to a TMQ aqueous suspension, a significantly higher drug release was observed in the nanoemulsions. A selected drug-loaded nanoemulsion was then incorporated into a hydrogel system, creating the intended semisolid dosage form, a nanoemulgel. To form the nanoemulgel, the selected nanoemulsion was uniformly dispersed into a Carbopol^®^ 940 gel matrix. This step aimed to create a final concentration of 0.5% TQM in nanoemulgel form, with the desired consistency, making it more suitable for topical application and ensuring a patient-friendly experience. The pH of the developed nanoemulgel was found to be within the range of skin’s acid mantle (5.53), making it suitable for topical use, ensuring compatibility with the skin, and minimizing potential irritation. Moreover, the prepared nanoemulgel exhibited a similar rheological behavior to a placebo gel, demonstrating pseudoplastic behavior, with thixotropic properties, which is desirable for topical application. It also demonstrated excellent spreadability, making it suitable for topical application on wounded skin, and with the spreading area increasing proportionally with the applied force. Drug skin permeability and deposition investigation was conducted in an ex vivo study, using a Franz diffusion cell, on excised skin from Wistar rats, comparing the developed nanoemulgel to a conventional gel formulation. For that study, a shaved and excised dorsal skin sample from a Wistar rat was placed between the donor and receptor compartments of the Franz diffusion cell. Then, 500 mg of the test formulation was placed in the donor compartment, and the receptor compartment was filled with phosphate buffer. At various intervals, 1 mL aliquots were withdrawn and replaced with fresh media. Subsequently, these aliquots were diluted and quantified using a UV-spectrophotometer to estimate drug permeation through time. Results confirmed the expected enhancement in drug permeation, with the TMQ nanoemulgel showing a significantly enhanced cumulative drug permeation (549.16 μg/cm^2^) when compared to the conventional gel form (120.75 μg/cm^2^). Also, the percutaneous drug flux of TMQ from the nanoemulgel was approximately five times higher (23.14 µg/cm^2^·h) than from the conventional gel form (4.78 µg/cm^2^·h), as was the permeability coefficient (9.26 K × 10^−3^ vs. 1.91 K × 10^−3^). This enhanced permeation may be attributed to the presence of surfactant/cosurfactant mixture in the developed formulation, and to the small droplet size characteristic of the developed nanosystem. In addition, in order to estimate drug deposition in rat skin, the tape stripping technique was employed. After the 12 h ex vivo skin permeability study, the skin sample was removed from the assembly and washed with buffer. The first strips were discarded, and the subsequent 15 strips were used to remove the subcutaneous layer. The treated skin sample and the stripped tape were then chopped and incubated in ethanol to fully extract the drug. The incubated sample was sonicated and centrifugated before analyzing the extracted drug by using a UV-spectrophotometer, to determine the amount of drug deposited in the skin. The skin deposition of TMQ from the nanoemulgel form was significantly higher, measured to be 965.65 μg/cm^2^, compared to 150.93 μg/cm^2^ from the gel formulation. Moreover, the local accumulation efficiency of the nanoemulgel was higher by a factor of 1.4 when compared to the conventional gel, indicating a greater drug accumulation in the skin for localized and prolonged therapeutic action. Moreover, according to the in vivo studies performed in a Wistar rat wound model, the application of the nanoemulgel on the wounds resulted in accelerated wound closure, evidenced by reduced wound size, enhanced re-epithelization, and increased collagen deposition, with higher efficacy than control groups (no treatment, standard 1% *w*/*w* silver sulfadiazine cream, and TMQ conventional gel) (Figure 5B). On the fourth day post-wounding, untreated rats displayed a hard thrombus swelling and exudates at the wound area. In contrast, animals from other groups exhibited a comparatively softer thrombus with reduced inflammation and no discharge. By the eighth day, reddish connective tissue, or granulation tissue, started forming in all groups. The complete epithelization time for the untreated group was 16.6 days. The groups with 1% silver sulfadiazine cream, TMQ conventional gel, and TMQ nanoemulgel had significantly shorter complete epithelization periods of 11.6, 14.33, and 10.33 days. A histopathological analysis (Figure 5C), on day 20 after treatment, revealed that the TMQ-nanoemulgel-treated group displayed larger amounts of granulation tissue and fewer mononuclear inflammatory cells compared to animals treated with 1% silver sulfadiazine and TMQ conventional gel. These findings suggest that the developed TMQ nanoemulgel has a significant wound healing potential, comparable to the 1% silver sulfadiazine cream, making it a promising formulation for topical wound healing applications. Thus, a topical TMQ nanoemulgel formulation was developed, by combining biocompatible polymers, oils, surfactants, and cosurfactants, resulting in a stable nanoemulgel with enhanced drug delivery potential and, hence, improved therapeutic efficacy, making it a promising formulation for potential use in dermatological applications, and specifically for improved skin wound healing.

**Figure 6:**
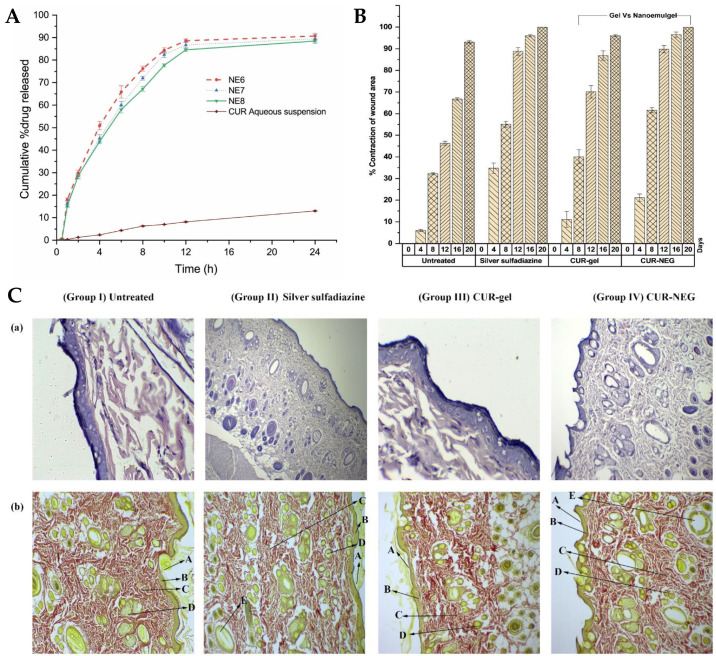
(**A**) In vitro cumulative drug release from the developed preliminary curcumin nanoemulsions, compared to the control (drug suspension); (**B**) in vivo wound-healing activity, in a rat model, of the developed curcumin nanoemulgel (CUR-NEG), compared to a curcumin conventional gel (CUR-gel), a marketed control formulation (Silver sulfadiazine), or no treatment (Untreated), including contraction of wound area percentage; (**C**) histopathology analysis of the rat’s skin tissue at day 20 after treatment, including indications for the *stratum corneum* (A), the papillary dermis (B), collagen fibers (C), sebaceous glands (D), and hair follicles (E), (a) Stained with hematoxylin-eosin; (b) stained with vangeison to observe collagen formation (at 10× magnification); adapted from Algahtani et al. [153], reproduced with permission from MDPI (Creative Commons CC BY 4.0 470 license).

Recently, natural compounds like curcumin have gained attention for their potential therapeutic effects on wound healing due to their anti-inflammatory, antioxidant, and antimicrobial properties. However, the clinical application of curcumin has been limited by its low solubility in aqueous media and poor skin permeability [192,193,194]. To tackle these issues, Algahtani et al. [153] developed a novel approach to enhance the wound healing potential of curcumin, through its formulation into a nanoemulgel, using the high-energy emulsification method of ultrasonication, known for its efficiency in producing nanosized droplets, in order to ensure uniform dispersion and stability of the nanoformulation. The first step involved the preparation of a preliminary O/W nanoemulsion, where curcumin was encapsulated within the oil droplets using Labrafac™ PG as the selected oil, and using a surfactant–cosurfactant system (Tween^®^ 80 and PEG 400). The droplet size of the curcumin-loaded nanoemulsion system was significantly influenced by the ratio of the Smix phase in the formulation components. Specifically, when the nanoemulsion system had a Smix ratio of 2:1, the droplet size was notably reduced, compared to the nanoemulsion system with a Smix ratio of 1:1. Additionally, the droplet size was found to be significantly influenced by the ultrasonication time. Specifically, 5 min of ultrasonication resulted in a notable reduction in droplet size, compared to only 3 min of ultrasonication, at constant Smix concentration and ratio. Additionally, the effect was more pronounced at a lower Smix concentration, rather than at higher ones. Nevertheless, the Smix ratio and ultrasonication time did not have a remarkable effect on the PDI. Selected preliminary nanoemulsions achieved a droplet size of less than 100 nm, with mean droplet sizes in a range from 49.61 to 84.23 nm, and PDI values from 0.10 to 0.23. Thermodynamic stress testing was also conducted on curcumin-loaded nanoemulsions, to assess their stability. The formulations exhibited high stability under heat–cooling cycles, freeze–thaw cycles, and centrifugation. This stability was attributed to the reasonably high zeta potential values, ranging from −15.96 ± 0.55 mV to −20.26 ± 0.65 mV, which minimized droplet coalescence and physical instability. The viscosity of the selected preliminary nanoemulsion systems was also evaluated, at room temperature, by rotational viscosimeter, with the results ranging from 83.74 mPas to 89.82 mPas. The difference in viscosity values was attributed to the increased concentration of surfactant between formulations (a reasonably viscous component). A UV-visible spectrophotometric analysis confirmed that the selected nanoemulsion formulations had high curcumin content, ranging from 98.86% to 99.23%. Thus, these formulations showed high drug encapsulation. With droplet sizes around 50 nm and desirable surface charges, the selected nanoemulsions were chosen for further investigation, as they were proven ideal for topical application, enabling improved skin permeability and deeper penetration. Hence, in vitro drug release was assessed, with all nanoemulsions being able to release up to 85% of curcumin within the first 12 h, while the release from aqueous suspension (control) was only around 10% at the same time. The small droplet size of the nanoemulsions could have been a critical factor in positively influencing the in vitro drug release, since it produces a large surface area for drug diffusion and, potentially, absorption to occur. The next step involved incorporating the curcumin nanoemulsion into a Carbopol^®^ 940 gel matrix, in order to form the curcumin nanoemulgel. The purpose was that this combination could take advantage of the benefits of both nanoemulsion and gel systems, both the high drug-loading capacity and improved skin permeation of nanoemulsions, and the enhanced stability and ease of application of gels. The drug concentration achieved in the final nanoemulgel preparation was 0.5% *w*/*w* of curcumin, and the formulation exhibited a favorable physicochemical profile, with a measured gel strength of 46.33 s, while the placebo gel system had a strength of 44.66 s. Additionally, the drug content uniformity of the curcumin nanoemulgel was calculated, showing a uniform dispersion of curcumin within the hydrogel, with a uniformity of 98.93%. Furthermore, the curcumin nanoemulgel exhibited a similar rheological profile to the placebo gel, with the incorporation of curcumin not affecting its rheological behavior, and exhibiting a thixotropic behavior, which is desirable for topical pharmaceutical dosage forms. Hence, the developed nanoemulgel proved to have the desired consistency for patient-friendly topical use, which aligned with small droplet size, appropriate zeta potential, a pH within the acceptable range for skin application, and optimal drug release properties, suggested its safe and effective application for wound healing. This adequacy for skin application was confirmed by the ex vivo skin permeation study results, conducted on excised rat skin, on Franz diffusion cells, to assess the ability of the curcumin nanoemulgel to penetrate the skin barrier. The results showed improved skin permeation of curcumin from the nanoemulgel formulation, further confirming its potential as an effective topical delivery system for wound healing applications. The cumulative amount of curcumin which permeated through the skin from the nanoemulgel was 773.82 μg/cm^2^, versus only 156.90 μg/cm^2^ from the conventional gel formulation. In addition, the curcumin nanoemulgel showed a six-fold increase in percutaneous drug flux compared to the conventional curcumin gel. Similarly, the permeability coefficient from the curcumin nanoemulgel also increased approximately six-fold when compared to the conventional gel. Furthermore, the permeation enhancement ratio and local accumulation efficiency of curcumin from the nanoemulgel were significantly higher than from the gel, with a shorter lag time (0.75 h versus 2.37 h). Moreover, the in vivo wound-healing activity (Figure 6A,B) from the nanoemulgel and gel formulations were evaluated and compared to a commonly used silver sulfadiazine formulation. The topical application of these formulations was performed and monitored, in Wistar rats, for 20 days. The results showed that all treated groups exhibited significant wound-healing activity compared to the untreated group. The curcumin nanoemulgel demonstrated almost equivalent wound-healing activity to the silver sulfadiazine gel, with both formulations leading to almost complete wound healing at day 20. Moreover, the histopathological evaluation (Figure 6C) confirmed the enhanced wound-healing activity of curcumin from the nanoemulgel formulation, showing reduced inflammatory cells and extensive collagen fiber production.

### 2.2. Skin and Skin Appendage Infections

**Figure 7:**
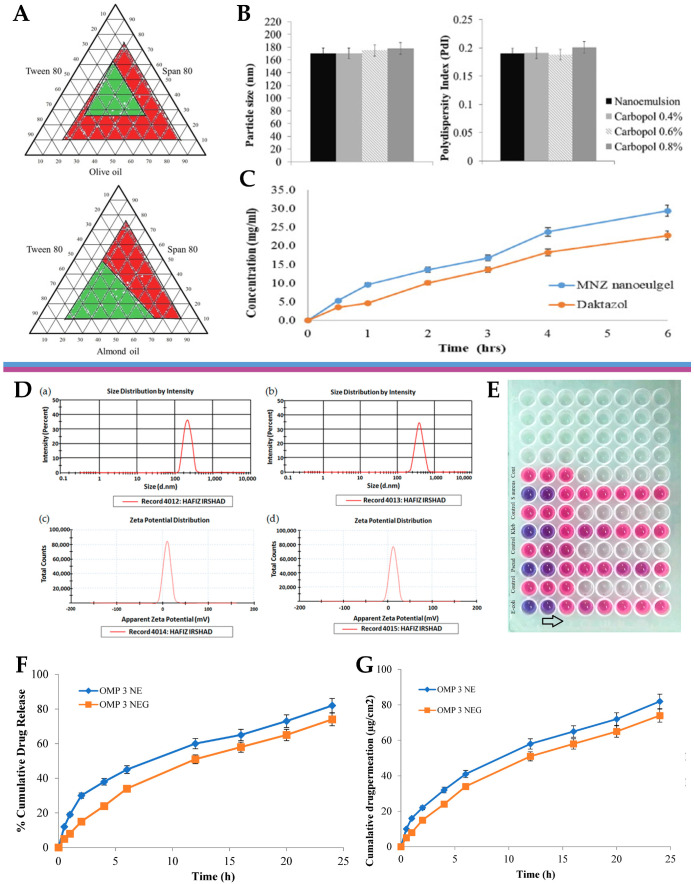
(**A**) Ternary phase diagrams of the preliminary nanoemulsions containing either olive oil, Tween^®^ 80, and Span^®^ 80, or almond oil, Tween^®^ 80, and Span^®^ 80; (**B**) droplet size and polydispersity index of the developed miconazole nitrate preliminary nanoemulsion and nanoemulgel formulations; (**C**) in vitro drug release profiles, in Franz diffusion cells, of the developed miconazole nitrate nanoemulgel, compared to the marketed product, Daktazol^®^ cream; adapted from Tayah et al. [154], reproduced with permission from Elsevier (license number 5671991093263); (**D**) droplet size (a) and zeta potential (c) of the developed omeprazole-loaded nanoemulsion, and droplet size (b) and zeta potential (d) of the developed omeprazole-loaded nanoemulgel; (**E**) minimum inhibitory concentration determination assay of the developed omeprazole-loaded nanoemulgel, against selected bacterial strains, using a 96-well microplate (arrow shows decrescent antimicrobial activity); (**F**) cumulative drug release percentage from the developed omeprazole-loaded nanoemulsion and nanoemulgel formulations; (**G**) cumulative drug permeation from the developed omeprazole-loaded nanoemulsion and nanoemulgel formulations; adapted from Ullah et al. [155], reproduced with permission from MDPI (Creative Commons CC BY 4.0 470 license).

The escalating global health threat posed by drug-resistant microbial and fungal infections necessitates the development of innovative and effective therapeutic strategies [195,196]. In this context, miconazole nitrate is a broad-spectrum antifungal medication commonly used to treat various fungal infections, especially on the skin. However, its therapeutic efficacy can be limited by factors such as poor drug permeation and low bioavailability [197,198]. Hence, a study by Tayah et al. [154] aimed to address these limitations by formulating miconazole into a nanoemulgel for topical application. Formulation composition was determined by selecting the excipients in which miconazole was most soluble, by dissolving it in various oils and surfactants. It was observed that olive oil and almond oil had the highest solubility, and among the surfactants, Tween^®^ 80 and Span^®^ 80 demonstrated the greatest ability to solubilize the drug. These oils and surfactants were selected as the drug vehicle, and hence preliminary O/W nanoemulsions were produced, using the self-emulsification technique. Optimized nanoemulsion formulations were chosen based on a ternary phase diagram (Figure 7A), according to which it was evident that the almond-oil-based formulations displayed the smallest particle size (170 nm) and PDI values (0.193). Therefore, the formulation containing almond oil was selected for subsequent experiments. Miconazole nanoemulgel formulations were then prepared by incorporating the preliminary miconazole nanoemulsions into a Carbopol^®^ 940 hydrogel, with Carbopol at different concentrations. The particle size and PDI remained consistent when the transformation of the nanoemulsion into nanoemulgels occurred (Figure 7B), with the three Carbopol concentrations (0.4%, 0.6%, and 0.8%) showing similar behavior, with particle sizes ranging from 170 to 180 nm. The zeta potential results confirmed the potential stability of the nanoemulgel formulations, with values just below −30 mV. Regarding the rheological properties of the nanoemulgel, its viscosity decreased with an increase in the shear rate, which indicated pseudoplastic behavior. The release of the drug was evaluated and compared to a market product, using the dialysis method. The results showed an inverse relationship between Carbopol concentration and release profile, which is in accordance with an increased viscosity. Hence, the formulation with the lowest Carbopol concentration (0.4%) exhibited the highest cumulative drug release. Furthermore, another in vitro drug release assay was also performed, this time using a Franz cell diffusion test (Figure 7C), to measure the cumulative drug release from the selected miconazole nanoemulgel (0.4% Carbopol) and compare it to a conventional marketed Daktazol^®^ cream (same drug molecule). After 6 h, the developed miconazole nanoemulgel exhibited a cumulative drug release of 29.67%, while the conventional Daktazol^®^ cream achieved a release of 23.79%. Hence, the developed nanoemulgel achieved a higher drug release within the studied timeframe, while still retaining a controlled release profile. Then, the antifungal activity of the developed miconazole nitrate-loaded nanoemulgel was evaluated against selected fungal strains, and its performance was also compared to that of the conventional gel formulation. The antifungal activity was assessed by conducting an agar-based test on *Candida albicans*, and the size of the inhibition zone was measured as an indicator of effectiveness. The miconazole nanoemulgel demonstrated the highest activity, with an inhibition zone of 40.9 ± 2.3 mm, showing significant improvements in antifungal activity when compared to the marketed formulation, hence suggesting a promising approach to overcome the limitations of current conventional gel formulations. These findings support the conclusion that the developed novel miconazole nanoemulgel formulation exhibited improved antifungal activity, as well as increased but controlled drug release, and other desirable characteristics, compared to the conventional cream. Nevertheless, further research will be required to evaluate the safety and in vivo efficacy of this novel formulation, and to address regulatory considerations for its potential clinical use. Yet, the development of this miconazole nitrate-loaded nanoemulgel formulation shows promising results in enhancing the antifungal activity of miconazole, with improved drug delivery properties, presenting a potential solution to enhance the therapeutic efficacy of miconazole nitrate in the treatment of skin fungal infections.

In another study, Ullah et al. [155] aimed to exploit the intrinsic antimicrobial properties of omeprazole, while utilizing chitosan’s unique properties to enhance drug delivery and targeting capabilities. In this context, the combination of omeprazole, a widely used proton pump inhibitor with known antimicrobial properties, and chitosan, a natural biopolymer with remarkable biocompatibility and mucoadhesive characteristics, holds significant promise [199,200]. Hence, the fabrication and comprehensive characterization of a novel omeprazole-based chitosan nanoemulgel formulation, intended for potential antimicrobial application, was performed. A preliminary O/W nanoemulsion was previously produced, with an oil phase consisting of olive oil and Span^®^ 80, and where omeprazole was solubilized. The aqueous phase was prepared by dissolving Tween^®^ 80 in distilled water. Then, both phases were mixed, with the oil phase being gradually added dropwise to the aqueous phase, and with both phases being heated and stirred. The mixture was then gradually cooled, still with continuous stirring, to ensure thorough homogenization and formation of a homogenous nanoemulsion. Different nanoemulsions were prepared, with varying concentrations of constituents, for optimization purposes. Then, the optimized nanoemulsion was transformed into a nanoemulgel by replacing the aqueous phase with a chitosan solution (0.1% *w*/*w*) prepared in 1% acetic acid. The optimized preliminary nanoemulsion showed a high entrapment efficiency of 81.36 ± 1.98%, while the resulting nanoemulgel exhibited similar values, with an entrapment efficiency of 78.23 ± 3.76%. The optimized nanoemulgel exhibited a droplet size of 369.7 ± 8.77 nm, and a PDI of 0.316, with the zeta potential values of the optimized drug-loaded nanoemulsion and nanoemulgel formulations being −11.2 ± 5.4 mV and −15.3 ± 6.7 mV, respectively, indicating a potentially relevant physical stabilization of the system due to the electrostatic repulsion between the droplets (Figure 7D). In the rheological analysis, the nanoemulgel exhibited higher viscosity compared to the nanoemulsion, which was to be expected, being attributed to the presence of the highly viscous Carbopol gel base, making it beneficial for the application of the formulation on the skin. Additionally, both the nanoemulsion and nanoemulgel formulations demonstrated excellent spreadability and extrudability, ensuring convenient application. Moreover, the pH values of the optimized nanoemulsion and nanoemulgel formulations remained consistently within the acceptable range of human skin pH, throughout an evaluation period of 38 days, highlighting their suitability for effective transdermal drug delivery. The overall stability of the optimized drug-loaded nanoemulgel formulation and the changes in particle size during storage were also assessed. The formulation’s appearance and clarity remained constant throughout the stability testing, and there was no change in the nanoemulgel’s particle size during storage at room temperature. However, under accelerated storage conditions, the particle size progressively increased from 369.7 ± 8.77 nm to 405 ± 9.65 nm, 480 ± 8.87 nm, and 529 ± 9.41 nm, at the end of the first, second, and third months, respectively. This observation indicates that higher storage temperatures led to a rise in particle size. Hence, while the optimized nanoemulgel formulation demonstrated stability under standard storage conditions, it experienced particle clustering when exposed to elevated temperatures. During the in vitro drug release analysis (Figure 7F), the nanoemulsion demonstrated a rapid initial release of omeprazole, with approximately 85.28% of the drug being released after 24 h. Conversely, the nanoemulgel exhibited a slightly lower initial burst release, with approximately 82.16% of the drug being released after the same time period. Hence, both formulations displayed a controlled drug release profile, but with high cumulative value, ideal for topical application. The study also aimed to evaluate the skin permeation capabilities of both the nanoemulsion and nanoemulgel formulations, a factor that is closely related to the preparations’ potential efficacy for transdermal drug administration. To assess this parameter, an ex vivo permeation assay was conducted (Figure 7G), using rabbit skin, providing insights into the drug permeation profiles of these formulations. The results revealed that the nanoemulsion demonstrated a higher permeation (82.18 ± 1.66 µg/cm^2^) when compared to the nanoemulgel (72.21 ± 1.71 µg/cm^2^), but this fact could simply be related to the higher viscosity of the nanoemulgel, which can be beneficial where formulation retention at the application site is concerned. Moreover, the success of transdermal drug permeation is influenced by several physicochemical properties, including particle size, zeta potential, and surface area. These factors play a pivotal role in determining the diffusion rate and the formulation’s ability to penetrate the skin effectively. Since both formulations have similar composition, in this context the surfactant Tween^®^ 80 likely played a vital role, by inducing lipid packing fluidization and optimizing the aqueous content in the *stratum corneum*, through a skin lipid extraction method. Additionally, Span^®^ 80, as a cosolvent and cosurfactant, also exerted an impact on drug permeation, contributing to these formulations’ favorable permeation profile. Furthermore, the combined effect of these formulations’ components probably reduced the epidermis’ barrier functions, providing the formulations with an advantage in promoting transdermal drug delivery. Additionally, the optimized nanoemulgel formulation displayed superior antibacterial effects compared to nanoemulsion (Figure 7E). The nanoemulgel exhibited reduced minimum inhibitory concentration (MIC) values against both Gram-negative bacteria (*Escherichia coli*, *Klebsiella pneumoniae*, *Pseudomonas aeruginosa*) and Gram-positive bacteria (*Staphylococcus aureus*). The formulation’s excipients and unique characteristics likely also contributed to its enhanced antibacterial activity. This improved effect was attributed to the nanoemulgel’s ability to transiently open tight junctions on the bacterial membrane, thereby increasing its antibacterial efficacy. Additionally, the presence of unsaturated fatty acids such as lactic acid in the formulation, further contributed to its antibacterial properties, by causing bacterial cell membrane rupture and eventual lysis. Therefore, overall, the nanoemulgel demonstrated promising potential as an effective strategy for controlling bacterial growth and promoting rapid healing. This is particularly critical in preventing the persistence of bacterial strains in injured skin tissues. In conclusion, the developed omeprazole-loaded chitosan nanoemulgel formulation represents a promising advancement in the field of antimicrobial drug delivery, offering new opportunities to combat microbial infections effectively and contribute to improved patient outcomes, while holding significant potential for therapeutic benefits in targeted drug delivery through the skin.

**Figure 8:**
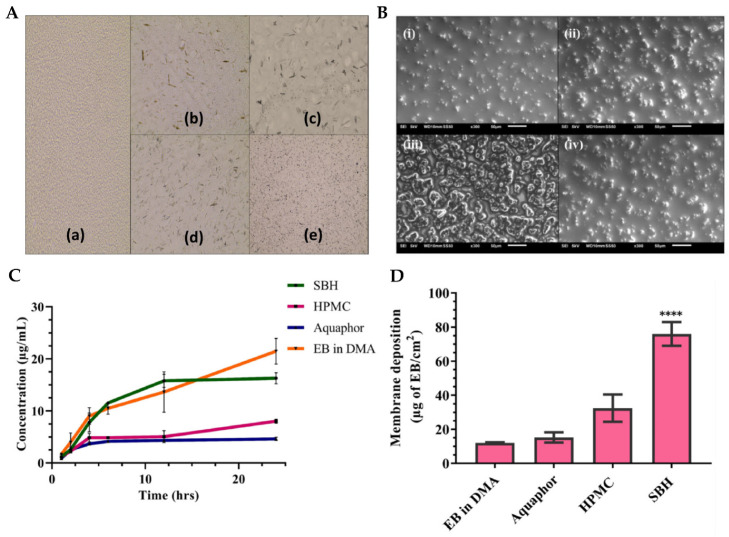
(**A**) Optical microscopy images of the EB-loaded preliminary nanoemulgels, containing either Soluplus^®^ (a), HPMC (b), Poloxamer 407 (c), Carbopol^®^ 974P (d), or Aquaphor (e); (**B**) scanning electron microscopy images of the optimized nanoemulgels, either drug-loaded Soluplus^®^ formulation (i), Soluplus^®^ vehicle (ii), drug-loaded HPMC formulation (iii), or HPMC vehicle (iv); (**C**,**D**) in vitro cumulative drug release (**C**) and membrane drug deposition (**D**) of the different EB-loaded nanoemulgels; **** *p* < 0.0001; DMA—dimethylacetamide; EB—Ebselen; HPMC—hydroxypropyl methylcellulose; SBH—Soluplus^®^; adapted from Vartak et al. [156], reproduced with permission from Elsevier (license number 5672000024708).

Fungal infections pose a substantial public health concern, affecting a significant number of individuals globally. Conventional antifungal therapies often encounter challenges such as drug resistance and limited efficacy, and hence the need for the exploration of alternative treatment strategies arises [201,202,203]. In this context, another study, by Vartak et al. [156], focused on the development and characterization of a novel Ebselen nanoemulgel, intended for the effective treatment of topical fungal infections. Ebselen (EB) is a well-established antioxidant and anti-inflammatory synthetic compound; nevertheless, it has limited solubility in commonly used solvents [204,205,206]. But since it has been proven to have reasonable solubility in dimethylacetamide (DMA), this cosolvent was selected to be part of the formulation’s composition. The rest of the excipients were also selected on a highest drug solubility basis, with Kolliphor^®^ ELP being selected as surfactant, and medium chain triglycerides (Captex^®^ 300 EP/NF) as oil phase. Additionally, in order to prevent drug precipitation and enhance formulation stability, a gelling polymer mixture was added to the external phase, hence producing an O/W nanoemulgel. Different polymers and polymer combinations were evaluated. This was achieved by mixing the components in a 5:7 ratio of oil to surfactant, and then mixing the resulting nanoemulsion in a 1:1 ratio with the gel bases, to create the final nanoemulgels. Hydroxypropyl methylcellulose (HPMC) K4M and Aquaphor, both present at a concentration of 0.5% *w*/*w*, formed a clear system when combined with 1 mg of EB in DMA. However, an increase in EB loading to approximately 2 mg caused immediate precipitation of EB. Similarly, when EB was added to a Carbopol^®^ 974P and Poloxamer 407 gel, at different gelling concentrations and loading levels, precipitation of the drug occurred. In contrast, the nanoemulgel prepared using Soluplus^®^ showed a delayed precipitation effect. This polymer was also proven to be an effective solubilizer for EB, hence playing a crucial role as both a solubility and drug-loading enhancer, and a formulation viscosity enhancer and stabilizer. Optical microscopy (Figure 8A) and scanning electron microscopy (SEM) (Figure 8B) images of EB-loaded nanoemulgels supported the stability of the Soluplus gel, when compared to all other gel compositions, not showing any drug precipitation. In contrast, the HPMC K4M nanoemulgels displayed distinct precipitation of EB throughout the system, manifesting as large, irregularly shaped crystals with small oil globules. In the optimized Soluplus^®^ spontaneously formed nanoemulgel, EB was present at 1% *w*/*w*, and the nanosystem revealed a droplet size of 54.82 ± 1.26 nm and zeta potential of −1.69 mV. Furthermore, the findings of rheological studies showed that the optimized EB-loaded nanoemulgel displayed a non-Newtonian fluidic behavior. The observed decrease in viscosity with increasing rotational speed confirms the pseudoplastic behavior of the nanoemulgel, rendering it an ideal choice for topical application. In the in vitro drug release study (Figure 8C), HPMC K4M and Aquaphor nanoemulgels initially showed similar drug release, but after 24 h, the HMPC K4M gel exhibited a three-times higher drug release. Interestingly, the release profile of EB in a DMA solution was similar to that of the Soluplus^®^ nanoemulgel, which demonstrated approximately two- and four-times higher release than the HPMC and Aquaphor formulations, respectively, after 24 h. Additionally, the optimized nanoemulgel exhibited a controlled release profile. Furthermore, the membrane deposition study (Figure 8D), which quantified the drug entrapped within the used membrane, showed that the nanoemulgel prepared using Soluplus^®^ displayed a significantly higher drug deposition, compared to all other formulations, from 2.3- to 5-fold higher. Relating to the formulations’ antifungal activity, resazurin, a redox indicator, was employed to assess the viability of fungal organisms, more specifically, multi-drug-resistant *Candida albicans* and *Candida tropicalis*, in culture plates. In a 48 h study, conducted in RPMI media, the EB-loaded optimized nanoemulgel demonstrated potent antifungal activity, exhibiting an MIC of 20 μM, exhibiting higher efficacy than the control, terbinafine hydrochloride (100 μM), a known antifungal drug. In conclusion, the developed novel topical EB nanoemulgel revealed improved drug solubility, membrane permeability, and deposition, and even displayed substantial antifungal activity against *Candida* infections (with higher potency than terbinafine hydrochloride, a commonly used antifungal agent), making it a potentially more effective alternative than existing conventional treatments.

Mahtab et al. [157] also developed a novel formulation for fungal infection treatment, namely, a transungual delivery system utilizing a nanoemulgel with ketoconazole for the efficient management of onychomycosis, a prevalent fungal infection that affects the nails and presents significant challenges in terms of treatment effectiveness and patient adherence [207,208]. At first, different primary nanoemulsions were produced, using GRAS excipients, with Labrafac ™ Lipophile WL1349 (medium-chain triglycerides of caprylic (C8) and capric (C10) acids) as the oil phase (highest ketoconazole solubility capacity), Polysorbate 80 as the surfactant, and PEG 400 as the cosurfactant. The surfactant and cosurfactant were combined in varying weight ratios (1:0, 1:1, 1:2, 2:1, 3:1, 1:3, and 4:1), with increasing concentrations of either the surfactant or cosurfactant in relation to each other. After solubilization of the drug within it, the mixture of oil, surfactant, and cosurfactant was then added dropwise to the aqueous phase, under moderate agitation on a vortex mixer, to form the nanoemulsions. Subsequently, high-pressure homogenization was used to achieve a desirable droplet size, and the resulting products were visually evaluated for clarity, transparency, and flowability. The selected primary nanoemulsion, with a surfactant-to-cosurfactant ratio of 3:1, revealed a mean droplet size of 77.52 ± 0.92 nm, a PDI of 0.128 ± 0.035, a zeta potential of—5.44 ± 0.67 mV, a pH of 6.2 ± 0.34, and a viscosity of 20.00 ± 1.24 cP. Additionally, stability studies confirmed the physicochemical stability of the system for 3 months, with the optimized nanoemulsion only showing very negligible changes in the previously evaluated parameters, with no phase separation of flocculation being observed. From the optimized nanoemulsion, nanoemulgels were prepared by adding Carbopol^®^ Ultrez 21 as gelling agent, glycerin as humectant, methylparaben playing the role of antimicrobial agent, thioglycolic acid as a penetration enhancer, and aminomethyl propanol as a pH-adjusting agent, to achieve a pH in the range of 6 to 6.5. Different nanoemulgels were prepared, using a combination of high-pressure homogenization and ultrasonication techniques, with different ratios of Carbopol-to-thioglycolic acid: 0.5 to 1.0, 1.0 to 1.5, and 1.5 to 1.0. Among these variations, the 1.0 to 1.5 proportion was found to have the best visual appearance, being glossy, creamy, viscous, and having a smooth and homogeneous texture, with no signs of phase separation, and was therefore selected for further studies. The physicochemical properties of the optimized nanoemulgel were extensively characterized, including pH (6.4 ± 0.24), viscosity (1142 ± 10.33 cP), spreadability (3.5 ± 0.22 g cm/s), extrudability (1.4 ± 0.56 g/cm^2^), firmness (13450 ± 231 g), consistency (6133 ± 19 g s), cohesiveness (169 ± 2.23 g), and adhesiveness (20.4 ± 0.81 g). Additionally, in vitro drug release studies showed that the optimized nanoemulgel exhibited sustained release of ketoconazole from the nanoemulgel over time, suggesting its potential for prolonged therapeutic effectiveness. The observed sustained release pattern indicates that the formulation was able to release the drug gradually over time, which can be potentially beneficial for treatment outcomes, as it might ensure a continuous and prolonged presence of the antifungal agent at the site of infection. Moreover, ex vivo transungual permeation results, in a goat hooves model, showed that the optimized nanoemulgel demonstrated higher drug permeation (77.54 ± 2.88%) when compared to the primary optimized nanoemulsion (62.49 ± 2.98%) and an aqueous drug suspension (38.54 ± 2.54%) during a period of 24 h. Additionally, the nanoemulgel’s antifungal activity was assessed against clinical isolates of dermatophytes, namely, *Trichophyton rubrum* and *Candida albicans*, using the agar diffusion method. The ketoconazole-loaded nanoemulgel displayed superior antifungal activity when compared to a conventional gel, with a larger zone of inhibition, highlighting its potential for better therapeutic outcomes. The developed formulation’s skin irritation potential was also assessed on rats, with the nanoemulgel showing minimal skin irritation, with no significant signs of erythema or edema, compared to the positive control. In addition, the histopathological analysis showed no significant pathological changes, indicating its safety for topical application. Therefore, overall, the results suggest that the optimized nanoemulgel holds promise as an effective delivery system for nail fungal infection treatment.

**Figure 9:**
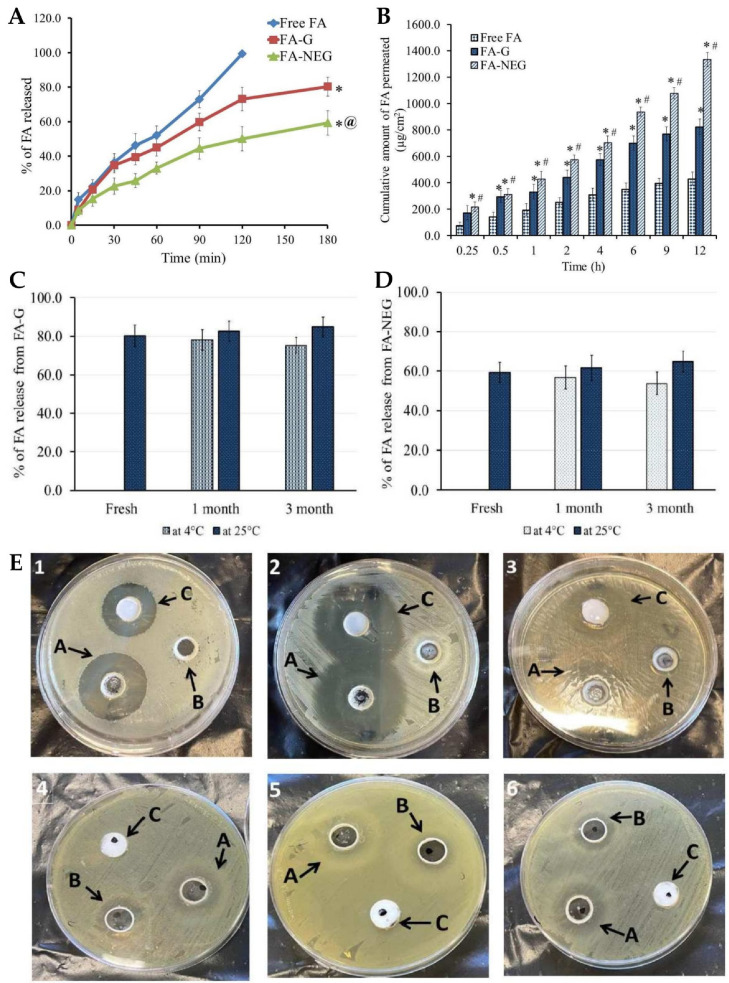
(**A**) In vitro FA release profiles from the developed nanoemulgel (FA-NEG), compared to a conventional gel (FA-G) and a drug suspension (Free FA), where * *p* < 0.05 compared to the drug suspension, and @ *p* < 0.05 compared to the conventional gel; (**B**) ex vivo FA permeation profiles, across rat skin, from the developed nanoemulgel (FA-NEG), compared to a conventional gel (FA-G) and a drug suspension (Free FA), where * *p* < 0.05 compared to the drug suspension, and # *p* < 0.05 compared to the conventional gel; (**C**,**D**) variation of the in vitro drug release during stability studies, under storage at 4 °C and 25 °C for 1 and 3 months, for the developed nanoemulgel (C) and conventional gel (D) formulations; (**E**) inhibition zone diameter photographs after treatment with the developed FA-loaded nanoemulgel (A), placebo nanoemulgel (B), or marketed FA formulation (C), on *Bacillus subtilis* (1), *Staphylococcus aureus* (2), *Enterococcus faecalis* (3), *Candida albicans* (4), *Shigella* (5), and *Escherichia coli* (6); FA—fusidic acid; adapted from Almostafa et al. [158], reproduced with permission from MDPI (Creative Commons CC BY 4.0 470 license).

Another study, by Almostafa et al. [158], also focused on the development of a novel approach to combat skin bacterial infections, using a nanoemulgel formulation containing fusidic acid (FA) and myrrh oil. FA is a potent antibiotic, effective against a panoply of Gram-positive bacteria, but it has poor water solubility, which poses a significant challenge for its effective formulation [209,210,211]. Myrrh oil, a traditional herbal extract, was used to modify and enhance the transdermal delivery of FA. In addition to these components, Tween^®^ 80 was used as a surfactant, Transcutol^®^ P as a cosurfactant, and CMC as a viscosifying and gelling agent. In initial studies, it was observed that increasing oil concentrations led to a relative increase in particle size for all preliminary nanoemulsions. Contrarily, a decrease in nanoemulsion particle size was observed with an increase in surfactant concentration, while keeping the oil concentration constant, due to a reduction in surface tension. The particle size of all developed preliminary nanoemulsions ranged from 116 to 226 nm. Moreover, the in vitro release of FA from the fabricated nanoemulsion formulations was evaluated, and results showed that the percentage of FA released from all nanoemulsion formulations ranged from 40.1 to 75.6%, exhibiting a controlled release profile. Increasing oil concentration resulted in a decrease in the percentage of FA released, due to the resulting larger particle size, since it provides a smaller surface area. On the other hand, increasing Tween 80 and Transcutol P concentrations led to a substantial increase in the percentage of FA release, which can be attributed not only to these excipients’ strong solubilizing ability, but also to the resulting smaller particle size. Hence, overall, the particle size of the formulations was revealed to have a crucial role in the drug release process, as systems with smaller particle sizes achieved maximum drug release. The preliminary nanoemulsion formulation was hence transformed into a nanoemulgel by adding CMC to the external phase, and this formulation was also characterized for relevant parameters. The pH value for the FA nanoemulgel formulation was found to be within an acceptable range at 6.61, making it potentially safe for topical application and minimizing the risk of skin irritation. The viscosity was also measured to assess the formulations’ rheological behavior, as this influences drug diffusion and in vitro release. The viscosity of the developed nanoemulgel was found to be 25265.0 cP, higher than that of the corresponding CMC gel formulation, 15245.0 cP, which was also measured for comparison purposes. Hence, both fell within an appropriate range for topical application. The spreadability, which determines whether the uniform application of the formulation on the skin is possible or not, was also determined. Results showed that the FA gel exhibited a spreadability of 40.5 mm, while the nanoemulgel formulation had a spreadability of 33.6 mm, both indicating excellent spreadability despite the observed difference between the two formulations. The formulations were also studied for stability during storage at 4 °C and 25 °C, for 1 and 3 months, and both showed nonsignificant variation in physical properties under all studied conditions, compared to their freshly prepared counterparts. The in vitro drug release (Figure 9A) of FA from the developed gel and nanoemulgel formulations was also evaluated and compared to an FA suspension. The results revealed that the drug was completely released from the suspension after 120 min, reaching 99.5% of cumulative release. In contrast, the FA gel and nanoemulgel formulations released 80.3% and 59.3% of FA after 180 min, exhibiting a more controlled release profile, as is intended for topically applied formulations. These results can be explained by the substantially increased viscosity of the gel and, especially, the nanoemulgel formulations, which slowed down the diffusion of the drug from the formulations and into and through the membrane. Additionally, the nanoemulgel formulation could have acted as a drug reservoir, where the drug passed from the inner phase to the outer one, further slowing the release rate. Furthermore, FA release from both preparations remained consistent during storage (Figure 9C,D), when compared to freshly prepared formulations. The results confirm the stability of the formulations and demonstrate the efficacy of nanoemulgels as nanocarriers. Skin permeability studies (Figure 9B) were also conducted, with the permeability of FA when incorporated into different formulations being evaluated using excised animal skin. The results further supported the potential of the developed formulations, since they showed that the FA nanoemulgel exhibited the highest permeability, followed by the FA gel, and only then the FA suspension, which exhibited the lowest skin permeability. This proven superiority of the nanoemulgel and gel formulations when compared to the drug suspension could be due to the incorporation of Transcutol P, a known effective permeation enhancer, especially effective in increasing permeation through the skin (more specifically, the *stratum corneum*), which certainly contributed to the significantly improved drug permeation profile through rat skin. The safety of the developed formulations was also tested, with the animals’ back skin being treated with the test formulations, and then undergoing a thorough examination to check for any sensitivity reactions. Fortunately, no signs of inflammation, irritation, erythema, or edema were observed on the inspected area during the entire 7-day study period. These results indicate that the formulations are potentially safe and well tolerated by the skin, without causing any adverse reactions or sensitivity issues. Moreover, the antibacterial activity of the developed FA nanoemulgel was evaluated (Figure 9E) against various microorganisms and compared to a placebo nanoemulgel and a common marketed cream. The FA nanoemulgel showed significant antibacterial activity against *Staphylococcus Aureus*, *Bacillus subtilis*, and *Enterococcus faecalis*, with a larger inhibition zone than the placebo or the marketed cream. Moreover, both the FA-loaded nanoemulgel and the placebo nanoemulgel exhibited significant inhibition zones against *Candida albicans*, *Shigella*, and *Escherichia coli*, while the marketed cream showed a negative effect against these bacteria. The combination of FA and myrrh oil in the developed nanoemulgel likely contributed to this enhanced antibacterial activity. In conclusion, FA was successfully incorporated into a nanoemulgel prepared with myrrh essential oil, showing good physical properties for topical application, enhanced skin permeation, no skin irritation, and potent antibacterial and antifungal activity, against several different types of microorganisms, with the combination of FA and myrrh essential oil showing synergistic effects. Hence, the results demonstrated that the developed nanoemulgel showed promise as a potential topical or transdermal drug delivery system for the treatment of skin bacterial or fungal infections, offering a new platform for innovative and effective topical treatments, and providing a basis for future research and clinical applications in dermatology.

### 2.3. Skin Cancer

**Figure 10:**
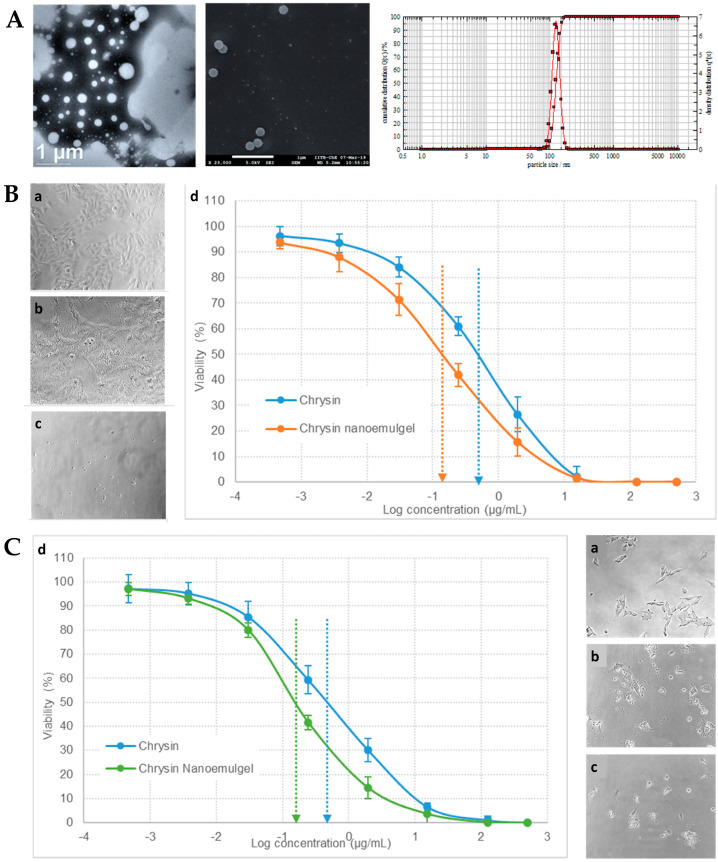
(**A**) Developed chrysin nanoformulation’s droplet size and size distribution, with transmission electron microscopy image (left), scanning electron microscopy image (middle), and photon cross-correlation spectroscopy results (right); (**B**) A375 cells’ morphological observation and growth inhibition after no treatment (control cells, a), treatment with chrysin solution (b), or treatment with the developed chrysin nanoemulgel (c), and respective in vitro cytotoxicity profile (cell viability %) (d); (**C**) SK-MEL-2 cells’ morphological observation and growth inhibition after no treatment (control cells, a), treatment with chrysin solution (b), or treatment with the developed chrysin nanoemulgel (c), and respective in vitro cytotoxicity profile (cell viability %) (d); adapted from Nagaraja et al. [142], reproduced with permission from MDPI (Creative Commons CC BY 4.0 470 license).

Skin cancer is a prevalent form of cancer with increasing incidence rates worldwide. Conventional treatment options often come with limitations, such as systemic side effects and inadequate skin permeation [212,213,214]. To fight these limitations, Nagaraja et al. [142] also developed a novel topical nanoemulgel formulation, incorporating nanosized droplets encapsulating the drug, chrysin, a flavonoid with proven anticancer potential, into a gel matrix for improved drug delivery and extended release, aiming for it to be an effective skin cancer treatment [215,216,217]. Stable lipid-based nanoemulsifying preconcentrates containing chrysin, made of Capryol^®^ 90 (oil and hydrophobic surfactant), Tween^®^ 80 (hydrophilic surfactant), and Transcutol^®^ HP (cosurfactant and cosolvent), were designed to be easily reconstituted in a gel base made of Pluronic^®^ F127 (gelling agent). The formulation components were selected in order to achieve the highest chrysin solubilization and, consequently, drug strength in the final formulation. Additionally, a surfactant mixture of 2:1 of Tween^®^ 80 to Transcutol^®^ HP was proven to be the best combination for highest solubilization. The physicochemical characterization of the nanoemulsifying drug delivery system showed its small particle size (Figure 10A), of less than 300 nm, a good homogeneity, with a PDI value around 0.26, indicating narrow droplet size distribution, and a negative zeta potential value, of about −15 mV, indicative of reasonable formulation stabilization potential. Moreover, the formulation was further stabilized by the incorporation of the oil droplets in a semisolid gel base. The chrysin-nanoemulsifying preconcentrate was added to water containing the gelling agent, Pluronic^®^ F-127, at 10 °C, for easier mixture and overall nanoemulgel preparation, due to this polymer’s thermosensitive properties. In addition, the droplet size and PDI values of the system did not change significantly after a 3-month storage period, hence ensuring its good stability under the studied conditions. Regarding the results of the performed ex vivo permeation studies (rat abdominal skin), while a chrysin conventional Pluronic^®^ gel base, used as control, showed poor penetration through the skin, the developed nanoemulgel exhibited a superior performance due to the presence of nanosized droplets, which resulted in better and faster skin permeation. In vitro cytotoxicity studies of the developed chrysin nanoemulgel were performed on various skin cancer cell lines, including A375 (human melanoma, Figure 10B), A375.S2 (human melanoma), SK-MEL-2 (human melanoma, Figure 10C), B16-F1 (murine melanoma), and A431 (human epidermoid carcinoma). Results showed that chrysin had matrix metalloproteinase-2 inhibitory activity, leading to a strong antiproliferative effect, with cell mobility and migration inhibition, with the developed nanoemulgel having a better performance than the control formulation, leading to deeper changes in cancer cell morphology. Furthermore, biocompatibility tests were also carried out, on L929 cells (noncancerous murine cells), which showed no changes in cell growth, therefore making the developed formulation safe for topical application. In conclusion, the incorporation of chrysin into a topical nanoemulgel formulation resulted in a significant enhancement of its therapeutic response in cytotoxicity studies, with the developed drug delivery platform technology exhibiting substantial advantages when compared to conventional formulations, such as its versatility, extended skin permeation, and retention, and the ability to potentially reduce systemic drug absorption.

**Figure 11:**
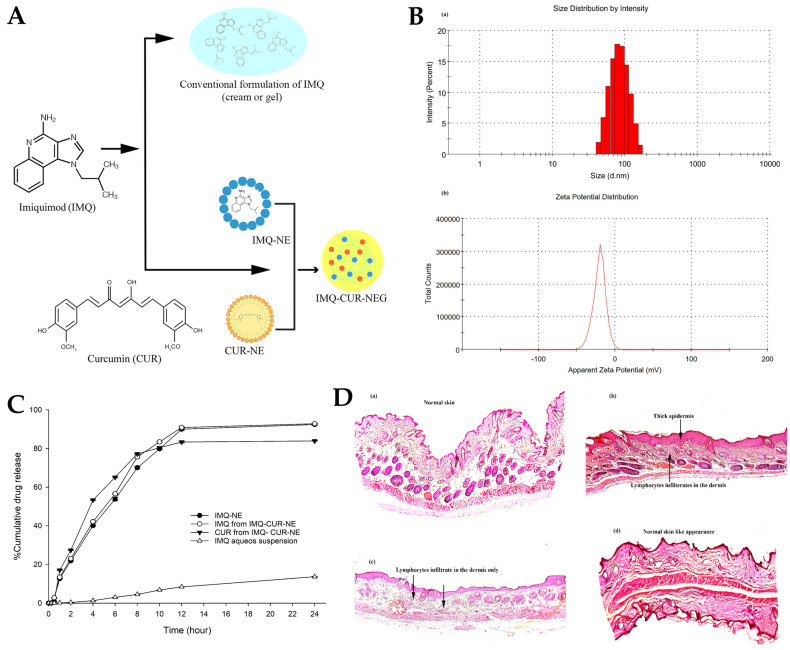
(**A**) Schematic representation of the developed IMQ-CUR-nanoemulgel; (**B**) droplet size (a) and zeta potential (b) distribution of the developed preliminary nanoemulsion; (**C**) in vitro IMQ and CUR drug release profiles from different formulations, including an IMQ-nanoemulsion (IMQ-NE, with no CUR), IMQ-CUR-nanoemulsion (with both IMQ and CUR), and an IMQ aqueous suspension (control); (**D**) histopathology images of the mice’s skin after the ten days of topical treatment, with either the IMQ gel (b), the IMQ-nanoemulgel (c), the IMQ-CUR-nanoemulgel (d), or no treatment (a); CUR—curcumin; IMQ—imiquimod; adapted from Algahtani et al. [159], reproduced with permission from MDPI (Creative Commons CC BY 4.0 470 license).

A different study, by Algahtani et al. [159], aimed to develop a novel formulation that combined the immunomodulatory effects of imiquimod (IMQ) and the anti-inflammatory properties of curcumin (CUR), for skin cancer treatment, using a nanoemulgel delivery system (Figure 11A). IMQ is a commonly used chemotherapeutic agent for skin cancer [218,219]. On the other hand, the combination with CUR has been shown to improve the therapeutic effectiveness of various chemotherapeutics, with this molecule also having intrinsic anticancer properties [220,221]. Nevertheless, the topical delivery of IMQ and CUR can be difficult due to their poor solubility and low skin penetration properties, and IMQ has been shown to lead to psoriasis-like lesions when applied topically [222,223]. Hence, the purpose of the incorporation of both IMQ and CUR into a topical nanoemulgel was to not only enhance drug permeation and provide sustained release of these drugs, but also reduce IMQ’s topical side effects, leading to improved therapeutic outcomes for skin cancer patients. For the selection of the most adequate excipients, IMQ solubility studies were performed at room temperature, and results showed that this drug’s solubility was maximized in oleic acid, in the oil category, which best mixed with Tween^®^ 20, in the surfactant category, and Transcutol^®^ HP, in the cosurfactant/cosolvent category, to form a stable nanoemulsion system through spontaneous emulsification, an advantageous low-energy method. Different ratio mixtures of oil and surfactant/cosurfactant were tested, and the different nanoemulsions were formed by addition of the aqueous phase. The preparation procedure included the accurate weighting of the necessary quantity of CUR and dissolving it completely in a homogeneous mixture of oil, surfactant, and cosurfactant, with the help of vortex mixing. Then, the aqueous phase was added, and the mixture was vortexed again. Clear, easily flowable, and transparent formulations were selected for further studies and characterized for relevant parameters. The analysis showed that the optically clear nanoemulsion formulations were composed of fine dispersed droplets in nanosized dimensions, ranging between 91.07 and 98.88 nm (Figure 11B). An increase in oil droplet size was correlated with an increase in oleic acid concentration and a decrease in surfactant/cosurfactant concentration. The zeta potential of the droplet surface was negative, ranging from −10.9 to −35.8 mV (Figure 11B), due to the presence of anionic groups in the fatty acids and glycols present in the nanoemulsions’ composition. This could be a potential advantage, as nanoemulsion droplets with a negative zeta potential tend to be more able to penetrate deeper in the skin. For the thermodynamic stability studies, the nanoemulsion formulations were subjected to stress conditions, namely, centrifugation, and heating–cooling and freeze–thaw cycles. Results confirmed their stability, since they did not show any physical changes such as phase separation, creaming, cracking, or coalescence. The final selected preliminary nanoemulsion formula, with a drug strength of 15 mg/mL, had a high percentage of drug content of 99.26%, a narrow droplet size distribution (10.57 nm) with a PDI of 0.094, a negative zeta potential of −18.7 mV, and a viscosity of 125.48 cP. The in vitro drug release for the optimized nanoemulsion was also performed (Figure 11C) using the dialysis bag technique. The release of IMQ from an aqueous suspension (control) was just 11% at 24 h, while the release from the preliminary nanoemulsion, containing IMQ only, was quite high, being around 92%. The incorporation of CUR into the nanoemulsion did not affect the release of IMQ, since the drug release from the formulation combining both drugs was 92.84% for IMQ and 83.94% for CUR. The selected nanoemulsion was then transformed into a nanoemulgel by incorporating Carbopol^®^ 934 as the gelling agent. The nanoemulgel had a mean droplet size of 78.39 nm and PDI of 0.254, which were still considered adequate for system stability and topical application. In spreadability studies, the spreading factors of the placebo nanoemulgel, the IMQ-loaded nanoemulgel, and the IMQ-CUR-loaded nanoemulgel were found to be equivalent, being equal to 0.82 cm^2^/m, 0.85 cm^2^/m, and 0.87 cm^2^/m, respectively. Hence, all formulations showed good extrudability potential from the container tube, adequate for patient-friendly applications. The overall drug content in the final nanoemulgel formulation was quite high, being around 99%, and the measured pH was 5.5, adequate in order to minimize skin irritation, since the ideal value is between 5 and 6. Ex vivo skin permeation and deposition studies were then performed in rat skin using Franz diffusion cells. Again, different formulations were compared, namely, the placebo nanoemulgel, the IMQ-loaded nanoemulgel, and the IMQ-CUR-loaded nanoemulgel. Formulations were introduced in the donor compartment, and the aliquots were taken at specified periods of time, filtered, and analyzed using UV-visible spectrophotometry. The tape stripping technique was used to determine the amount of drug deposited on the skin layer. The skin deposition of IMQ from the IMQ-nanoemulgel was 1205.2 μg/cm^2^, and 1367 μg/cm^2^ from the IMQ-CUR-nanoemulgel, which was 5 times higher than that obtained from a conventional gel formulation (control). CUR skin deposition was also around 9 times higher (5178 μg/cm^2^) in the developed IMQ-CUR-nanoemulgel than in the conventional gel formulation (570 μg/cm^2^). The percutaneous IMQ drug flux was also determined, being equal to 0.042 µg/cm^2^.h for the IMQ-nanoemulgel, and 0.071 µg/cm^2^.h for the IMQ-CUR-nanoemulgel, which was around 18 times higher than for the conventional gel formulation (0.004 µg/cm^2^.h). Hence, the developed nanoemulgel showed an improved permeation profile when compared to a conventional gel formulation, with the nanoemulgel containing both IMQ and CUR displaying apparent synergistic effects. In vivo studies were also performed, on mice, and skin pathological changes after 10 days of topical application of different formulations were monitored. Psoriasis-like symptoms started to appear on mice treated with the IMQ conventional gel formulation from the 2nd day of application and worsened until the 10th day. On the other hand, mice treated with the IMQ-nanoemulgel exhibited a delayed appearance of these symptoms, which was possibly correlated with a more controlled drug release from the formulation. Fortunately, the application of the IMQ-CUR-nanoemulgel did not lead to the appearance of psoriasis-like symptoms, which was connected with the antipsoriatic activity of CUR. Additionally, on the 11th day after the application of the formulations on the skin, the skin was collected for histopathology analysis (Figure 11D). While untreated skin showed regular epidermis and dermis, skin treated with the conventional gel showed hyperkeratosis, parakeratosis, acanthosis, and epidermal infiltrates. The IMQ-nanoemulgel treated skin showed comparable results, but less thickening of the epidermis layer, but the skin treated with the IMQ-CUR-nanoemulgel showed similar characteristics to untreated normal skin, with only a reduced number of infiltrates being observed. Hence, a stable nanoemulgel with optimal rheological properties, allowing easy spreadability on the skin, exhibiting high encapsulation efficiency for both IMQ and CUR, with sustained release profiles and enhanced permeation across the skin barrier, was successfully developed, while reducing psoriasis-like lesions in an animal model.

### 2.4. Skin Inflammatory Diseases

Psoriasis is a chronic inflammatory skin disorder affecting millions of individuals worldwide, being characterized by abnormal keratinocyte proliferation and inflammation. Current treatment options have limitations; hence, there arises the need for the development of novel and effective therapies [224,225,226]. In this context, another study, by Pund et al. [146], investigated the transcutaneous delivery of leflunomide, an immunomodulatory drug, using a nanoemulgel formulation for the treatment of both melanoma and psoriasis [227,228,229]. Based on leflunomide solubility studies, the excipients selected to form the preliminary O/W nanoemulsion were Capryol^®^ 90, Cremophor^®^ EL, and Transcutol^®^ HP, as oil base, surfactant, and cosolvent, respectively. These components were found to have good miscibility with each other, and the formed preliminary nanoemulsion was transformed into a nanoemulgel by incorporating it into a gel matrix made of Pluronic^®^ F-127. The preliminary nanoemulsions were evaluated for droplet size, which was found to be in the range of 98.7 to 280.92 nm, with a PDI between 0.2 and 0.3, indicative of narrow size distribution, and a slightly negative zeta potential of −7,8 mV. These characterization parameters were also measured in the nanoemulgel, with no significant changes being observed. Regarding the nanoemulgel’s viscosity, the shear thinning nature of the poloxamer gel was demonstrated by the decrease in viscosity at higher shear rates, which allows for effortless dispensing of the product from the container and smooth application on the skin. Additionally, the nanoemulgel demonstrated potent antipsoriatic activity by inhibiting the proliferation of human keratinocytes (HaCaT cell line) and reducing proinflammatory cytokine levels, such as interleukin-6 and tumor necrosis factor-alpha. The suppression of these proinflammatory cytokines is crucial in mitigating the inflammatory response associated with psoriasis. Moreover, the developed formulations also exhibited antipsoriatic activity by effect on leukocyte infiltration and keratinocyte proliferation. Furthermore, the nanoemulgel displayed significant antimelanoma activity by inducing apoptosis in A375 and SK-MEL-2 melanoma cells and inhibiting tumor cell proliferation. These effects were attributed to the cytotoxicity of leflunomide, which can target multiple signaling pathways involved in cancer growth and survival. Safety assays involved systemic biocompatibility assessment, by hemolytic toxicity evaluation, with results showing that the nanoemulgel had a minimal 0.25% hemolytic toxicity compared to the positive control (Triton-X), suggesting its compatibility with blood cells and potential for reducing the risk of adverse effects. In conclusion, the development and characterization of a novel leflunomide-loaded nanoemulgel was successful, with the results indicating that the developed formulation possesses favorable physicomechanical characteristics for transcutaneous delivery and exhibiting promising in vitro antipsoriatic and antimelanoma activity, suggesting its potential as a novel therapeutic approach for the treatment of these diseases.

**Figure 12:**
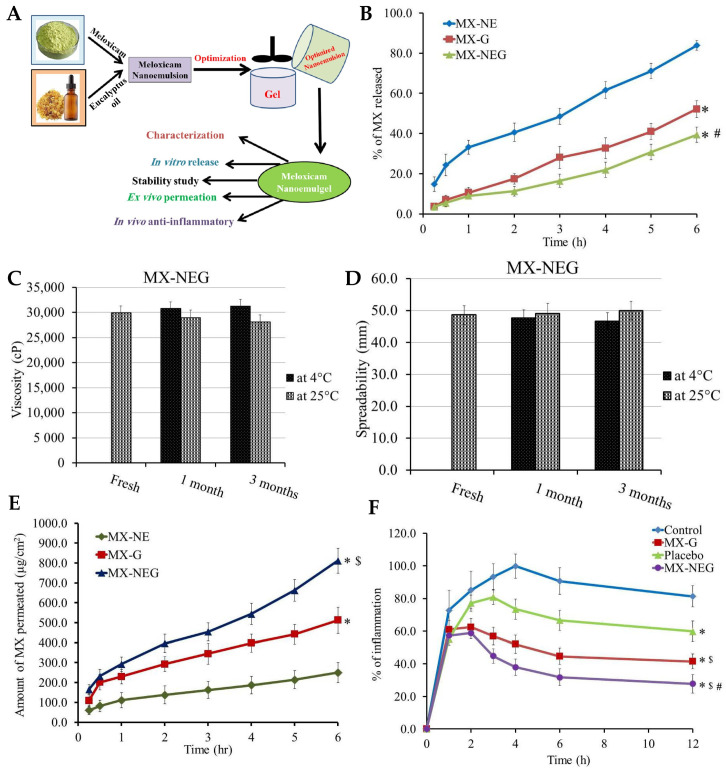
(**A**) Schematic representation of the developed MX and eucalyptus oil nanoemulgel, including performed physicochemical and efficacy characterization studies; (**B**) in vitro drug release profiles of the developed preliminary MX-nanoemulsion (MX-NE), conventional MX-gel (MX-G), and MX-nanoemulgel (MX-NEG), with * *p* < 0.05 compared to the preliminary MX-nanoemulsion, and # *p* < 0.05 compared to the conventional MX-gel; (**C**,**D**) stability profiles of the developed MX-nanoemulgel formulation, after 1 and 3 months, under storage at 4 °C and 25 °C, in what concerns viscosity (C) and spreadability (D); (**E**) ex vivo drug permeation profiles of the developed preliminary MX-nanoemulsion (MX-NE), conventional MX-gel (M-G), and MX-nanoemulgel (MG-NEG), with * *p* < 0.05 compared to the preliminary MX-nanoemulsion, and $ *p* < 0.05 compared to the conventional MX-gel; (**F**) anti-inflammatory effects of various formulations on rat hind-paw edema, including the developed MX-nanoemulgel (MG-NEG), a conventional MX-gel (M-G), a placebo formulation (nanoemulgel vehicle with no drug), and a control group (no treatment), with * *p* < 0.05 compared to the control group, $ *p* < 0.05 compared to the placebo group, and # *p* < 0.05 compared to the conventional MX-gel group; MX—meloxicam; adapted from Shehata et al. [160], reproduced with permission from MDPI (Creative Commons CC BY 4.0 470 license).

In another study, by Shehata et al. [160], the authors presented the development, characterization, and optimization of a novel eucalyptus-oil-based nanoemulgel loaded with meloxicam (Figure 12A) aimed at enhancing the anti-inflammatory efficacy of the drug for topical application. Nonsteroidal anti-inflammatory drugs, such as meloxicam (MX), have been widely used to alleviate inflammatory conditions; however, their application is often limited by issues like systemic toxicity and poor drug delivery to the targeted site [230,231]. To overcome these limitations, a nanoemulgel formulation was designed to facilitate controlled drug release and improve local drug concentration, as chronic inflammation is a critical pathophysiological process associated with numerous dermatological disorders with a strong inflammatory basis, hence necessitating effective and targeted therapeutic interventions. Therefore, in their research, several preliminary nanoemulsion formulations were developed by carefully blending specific ingredients. To create the oily phase, 1% (*w*/*w*) of MX was combined with a precise amount of eucalyptus oil and a cosolvent/cosurfactant, Transcutol^®^ P. For the aqueous phase, varying quantities of Tween^®^ 80 (as a surfactant) and PEG 400 (as a cosurfactant) were mixed with distilled water. The two phases were then meticulously merged, but achieving a well-mixed and stable nanoemulsion required high shear homogenization, so the formulation’s homogeneity was further enhanced by sonication. Additionally, the high solubility of meloxicam in the various components further confirmed their suitability as part of the formulation’s composition, with solubility values of 216 ± 11 mg/mL in eucalyptus oil, 130 ± 10 mg/mL in Tween^®^ 80, 68 ± 6 mg/mL in Transcutol^®^ P, and 146 ± 5 mg/mL in PEG 400. In the pursuit of optimal formulations, several nanoemulsions incorporating MX were developed, with different excipient proportions. Remarkably, all formulations exhibited excellent stability at room temperature, showing no signs of phase separation. The developed nanoemulsions displayed particle sizes within the nanometric range, ranging from 139 ± 2.31 to 257 ± 3.61 nm. Predictably, an increase in oil concentration led to larger particle sizes, likely due to a corresponding increase in the dispersed phase. Conversely, when the surfactant concentration was increased, while keeping the oil concentration constant, the formulation’s particle size decreased, a finding that aligned with previous studies, which reported an inverse relationship between nanoemulsion particle size and surfactant concentration. The results of the in vitro drug release studies (Figure 12B) performed on the preliminary nanoemulsions showed that the percentage of MX released from the NE formulations varied between 55.0 ± 2.8% and 87.6 ± 3.9% after 6 h, hence exhibiting a controlled release profile. Notably, increasing the oil concentration in the preparation led to a decrease in MX release, likely due to the larger particle size, resulting in a smaller surface area available for drug release. The optimized nanoemulsion formulation was then transformed into a nanoemulgel by integrating HPMC as the gelling agent. A conventional HPMC gel formulation, containing MX, was also developed, for comparison purposes. Both the MX-loaded nanoemulgel and the MX-gel formulation appeared smooth, homogenous, and physically stable, demonstrating drug distributions above 99.3%, indicating a uniform dispersion of the drug. Additionally, both formulations, and especially the MX-nanoemulgel formulation, showed suitable viscosity for easy skin application (Figure 12C) and satisfactory spreadability (Figure 12D). Moreover, stability assessment, over 1 and 3 months, showed nonsignificant variations in the MX-nanoemulgel formulation’s characterization parameters. In vitro drug release comparison revealed that the MX-nanoemulgel exhibited a lower percentage of drug release (39.4 ± 3.7%) compared to the MX-gel (52.1 ± 4.2%) and the optimized preliminary nanoemulsion (83.9 ± 2.41%), hence exhibiting a more controlled release pattern. This could be attributed to the incorporation of HPMC into the formulation’s external phase, resulting in a slower drug release due to increased viscosity. Nevertheless, the results of the ex vivo skin permeation study (Figure 12E), across rat skin, supported the superiority of the developed nanoemulgel when compared to the other formulations, since it was observed that after 6 h a significantly greater amount of MX permeated from the nanoemulgel formulation, with a steady-state transdermal flux value of 141.28 ± 9.17 μg/cm^2^, compared to the permeation from the MX-gel formulation, which showed a value of 84.28 ± 10.83 μg/cm^2^. The permeation of the MX-nanoemulgel was found to be enhanced by 1.68-fold when compared to the conventional MX-gel. This increased permeation of the drug from the nanoemulgel formulation can be attributed to its small particle size, leading to a larger surface area than the conventional gel formulation, since by incorporating the drug into nanosized globules its permeation through the skin layer is facilitated. Furthermore, the presence of Tween^®^ 80, PEG 400, and Transcutol^®^ P in the formulation also played a vital role in enhancing MX permeation from the nanoemulgel, due to these surfactants/cosurfactants’ capacity for increasing drug permeation through biological barriers. Moreover, according to the skin irritation tests performed on animals treated with the developed topical MX-nanoemulgel to ensure the safety of the formulation, no irritation, erythema, or edema were observed, indicating its safety. In addition, an in vivo anti-inflammatory study (Figure 12F), in which edema was induced in the rats’ hind-paw, was also performed. The thickness of the edema directly correlated with the percentage of inflammation. After 12 h, inflammation percentages were 81.3 ± 6.4%, 59.8 ± 6.4%, 41.5 ± 4.6%, and 27.8 ± 5.7% for the control group, placebo (nanoemulgel vehicle, with no drug), MX-gel, and MX-nanoemulgel groups, respectively. The placebo group’s reduced inflammation percentage, when compared to the control group, confirmed the anti-inflammatory effects of eucalyptus oil. Furthermore, the MX-nanoemulgel-treated group demonstrated a significantly higher anti-inflammatory effect when compared to the MX-gel group, suggesting the nanoemulgel’s greater anti-inflammatory effect due to improved skin permeability. Hence, the study provided evidence of eucalyptus oil’s anti-inflammatory effects, and its synergistic action with MX, making the developed nanoemulgel a promising option for treating inflammation-related skin conditions. In conclusion, an MX-loaded nanoemulgel was successfully developed and optimized, offering a promising nanocarrier topical formulation for enhanced drug delivery, good adhesion, and easy application onto the skin.

**Figure 13:**
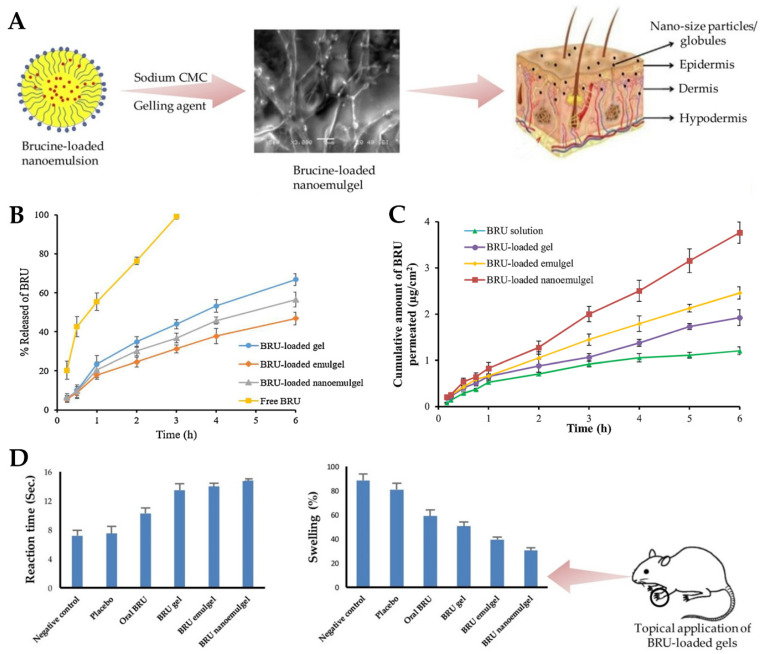
(**A**) Schematic representation of the developed brucine-loaded topical nanoemulgel, including a scanning electron microscopy image; (**B**) in vitro drug release profiles from different BRU-loaded formulations, namely, a drug solution (Free BRU), a conventional gel, an emulgel, and a nanoemulgel; (**C**) ex vivo drug permeation profiles from different BRU-loaded formulations, namely, a drug solution, a conventional gel, an emulgel, and a nanoemulgel; (**D**) antinociceptive (reaction time) and anti-inflammatory (swelling) effects of different brucine-loaded formulations after administration to mice; BRU—brucine; adapted from Abdallah et al. [161], reproduced with permission from Elsevier (license number 5672000458127).

A different study, by Abdallah et al. [161], focused on the preparation, characterization, and evaluation of a topical nanoemulgel containing brucine as an active substance (Figure 13A). Brucine is a natural alkaloid produced from *Strychnos nux-vomica* L. seeds. This plant-derived molecule has been used in traditional medicine to relieve arthritis and traumatic pain [232,233]. Aside from having antitumor and antiangiogenic activities, it has relevant anti-inflammatory and antinociceptive effects. Nevertheless, its clinical use is limited due to high lipophilicity, and consequent low water solubility, and to high toxicity, especially in oral administration [234,235,236]. Hence, the study aimed to develop a nanoemulgel containing brucine for anti-inflammatory and antinociceptive topical effects, hence increasing its targeted delivery and decreasing systemic side effects. A preliminary nanoemulsion was formulated first by mixing brucine with a suitable oil (myrrh oil), surfactant (Tween^®^ 80), cosurfactant (PEG 400), and cosolvent (ethyl alcohol), and then adding water. This formulation was then transformed into a nanoemulgel by incorporating it into a gel matrix, with sodium carboxymethyl cellulose as gelling agent, followed by a high-energy homogenization technique—high-pressure homogenization plus sonication. The droplet size of the developed nanoemulgel was evaluated and compared to that of an emulgel (same composition, but without high-energy homogenization), with results showing that the droplet size of the nanoemulgel (151 ± 12 nm) was 10-fold smaller than the droplet size of the emulgel (1621 ± 77 nm), hence showing significant improvement and displaying the relevance of the use of the high-pressure homogenization plus sonication in order to obtain the adequate nanometric size. The PDI values were also determined, which were 0.631 for the emulgel and 0.243 for the nanoemulgel, hence indicating monodispersity for the nanometric formulation (PDI value of less than 0.3), and further confirming the superiority of the nanoemulgel when compared to the emulgel formulation. Furthermore, a morphological evaluation of the nanoemulgel did not detect crystals of brucine, which indicated adequate drug solubility. Moreover, the brucine-loaded nanoemulgel showed adequate spreadability (48.3 ± 1.8 mm) and viscosity (62650 ± 700 cP) for topical application, comparable to those obtained for the emulgel and a conventional gel formulation (containing carboxymethyl cellulose). The in vitro drug release studies (Figure 13B) showed that brucine release from all developed formulations was lower and slower than that from a drug solution (99.5 ± 1.3%), hence exhibiting controlled release profiles. The amount of brucine released from the conventional gel formulation was greater (69.07 ± 2.54%) than that released from the emulgel (47.0 ± 4.32%) or nanoemulgel (58.6 ± 4.3%), probably due to a higher aqueous content in the gel formulation. Furthermore, stability studies showed that there were no changes in the color, homogeneity, spreadability, viscosity, or in vitro drug release of the nanoemulgel under the studied conditions (4 °C and 25 °C), hence making the developed formulation highly stable. Nevertheless, despite having a sustained drug release, the developed nanoemulgel formulation showed a superior drug permeation to all other formulations in ex vivo rat skin permeation studies using Franz diffusion cells (Figure 13C). Results showed that the best permeation through the skin was in fact attributed to the brucine-loaded nanoemulgel, and the worst to the brucine solution. Additionally, the cumulative amount of drug permeated from the nanoemulgel was substantially higher than from the other tested dosage forms. Moreover, the anti-inflammatory effects of the brucine-loaded nanoemulgel were evaluated using a carrageenan-induced paw edema animal model (Figure 13D). The animals were treated with the nanoemulgel formulation, and the degree of paw swelling was measured. Results showed a significant increase in the inflammation of the control and placebo groups after 4 h. The group treated with an oral drug solution reached the highest inflammation after 3 h, although it was lower than the control or placebo groups. Additionally, there were no significant differences between the brucine-loaded topical gel application and oral administration groups. Nevertheless, the brucine-loaded topical nanoemulgel group showed maximum inflammation after 1 h of treatment, with a remarkable decrease in swelling after 12 h. This result could be attributed to the nanoscale size of the nanoemulsion and the high penetration of the nanoemulgel through rat skin. Hence, a significant reduction in paw edema compared to the control groups indicated potent anti-inflammatory activity of the developed nanoemulgel. The antinociceptive effects of the developed formulations were also assessed using the acetic-acid-induced writhing test and hot plate test (Figure 13D). In the acetic-acid-induced writhing test, the formulations were administrated to the animals and then the number of abdominal writhing movements recorded. A reduction in writhing movements indicated the antinociceptive potential of a given formulation, and results showed that the developed nanoemulgel led to a significant reduction in the number of abdominal writhing movements, leading to a greater inhibition than all other groups. In the hot plate test, the animals were placed on a hot plate, at a temperature of 55 °C. Then the time that elapsed from when the animal was put on the hot plate until any reaction was produced was measured. Antinociceptive effects, produced from formulation administration, will increase the time until the animals react. Results showed that the maximum antinociceptive effect by oral administration was reached after 1.5 h and after 3 h for topical application. The brucine-loaded nanoemulgel showed greater antinociceptive effects than gel and emulgel dosage forms, which, again, can be due to the larger surface area and nanosized particles, which improve the penetration of brucine through the *stratum corneum*, and hence enhance its absorption to reach better antinociceptive effect. Skin irritation studies were also performed, in order to assess the safety of the developed formulations. Sensitivity reactions, such as edema or erythema, were monitored in the animals at 1, 8, and 24 h post-treatment. Results showed that there were no signs of erythema or edema after 24 h of treatment with the different brucine formulations, hence meaning that they were in fact well-tolerated and not skin-sensitizing. Therefore, the developed brucine-loaded nanoemulgel may be a safe and well-tolerated drug delivery vehicle for topical application, also having high efficacy in permeating the skin, and producing anti-inflammatory and antinociceptive effects.

### 2.5. Other Applications: Neuropathy and Antiaging Effects

Diabetic neuropathy is a common complication of diabetes characterized by nerve damage that leads to pain, numbness, and tingling sensations [237,238,239]. Capsaicin, a natural compound found in chili peppers, has shown promise in alleviating neuropathic pain [240,241,242]. The analgesic effect of capsaicin is attributed to its potential for activation of transient potential vanilloid type 1 receptors, leading to the depletion of inflammatory neuropeptides and neuronal desensitization [240,243,244]. Additionally, capsaicin exhibits anti-inflammatory properties by inhibiting the activity of proinflammatory mediators [245,246,247]. Nevertheless, despite its efficacy, the clinical use of capsaicin through oral or intravenous administration has limitations due to its short half-life, pungency, and first-pass metabolism, and noncompliance [248,249,250]. Moreover, its effective delivery through the skin remains a challenge. In this context, Saab et al. [162] aimed to optimize a nanoemulgel formulation for the transdermal delivery of capsaicin, evaluating its skin permeation properties and assessing its in vivo potential for diabetic neuropathy treatment. Initially, preliminary nanoemulsions were developed containing eucalyptus oil as the oil, Tween^®^ 80 as the surfactant, and propylene glycol, ethanol, and isopropyl alcohol as cosurfactants. Several different nanoemulsions were prepared, with different ratios of these excipients, and the formula exhibiting the best droplet size (28.15 ± 0.24 nm) and PDI (0.27 ± 0.05) values was selected for further studies, consisting of 8% oil phase, an oil to surfactant mixture ratio of 2.5-to-7.5 (Tween^®^ 80/cosurfactant mixture), and a final capsaicin concentration of 0.05%. Then, by combining the chosen nanoemulsion formula with a gel base, using Carbopol^®^ 940 as the gelling polymer, the nanoemulgel was successfully formed. The developed nanoemulgel was examined under transmission electron microscopy, alongside its corresponding nanoemulsion, with both showing spherical emulsion droplets within the nanosize range. Additionally, the emulsion droplets seemed to remain stable after gel incorporation, indicating resistance to destabilization processes, such as Oswald ripening, which was evident by it showing no significant changes in droplet size, PDI, transmittance percentage, or appearance after long-term storage (at 4 °C, 25 °C, and 32 °C), centrifugation, heating–cooling cycles, and freeze–thaw cycles. The rheological assessment of the nanoemulgel established its pseudoplastic behavior, with this formulation exhibiting a predictably higher viscosity (5580 mPa/s) than the correspondent nanoemulsion (11.5 mPa/s). The skin permeation study showed that the capsaicin-loaded nanoemulgel demonstrated enhanced (188.12 μg/cm^2^ cumulative permeation) and accelerated (0.19 μg/cm^2^.s^−1^ permeation flux) permeation compared to a conventional gel (46.38 μg/cm^2^ and 0.11 μg/cm^2^.s^−1^, respectively). The antinociceptive potential of the developed formulation was also evaluated, in an animal model, with the application of the capsaicin-loaded nanoemulgel having showed significant improvements in thermal latency, as demonstrated in the hot plate and tail withdrawal tests in alloxan-induced diabetic mice. Compared to the placebo-treated mice, the nanoemulgel treatment resulted in a remarkable increase in thermal latency in the 8th week. Conversely, treatment with conventional capsaicin gel showed less significant improvement. Furthermore, the capsaicin nanoemulgel exhibited superior efficacy in alleviating mechanical allodynia. The nanoemulgel treatment group showed a substantial improvement compared to the placebo-treated animals, approaching the level of improvement seen in the tramadol-treated group. These positive outcomes can be attributed to the successful transdermal delivery of capsaicin from the nanoemulgel, as previously confirmed in the skin permeation study. Hence, the optimized developed formulation exhibited favorable physicochemical properties and enhanced skin permeation, and the in vivo assays’ results demonstrated its efficacy in reducing pain-related behaviors and improving sensory function. Given this, the developed capsaicin-loaded nanoemulgel holds promise as a treatment option for diabetic neuropathy, offering a noninvasive, convenient, and effective approach to alleviate neuropathic pain. Further studies, including clinical trials, are necessary to validate its safety, efficacy, and long-term effects in human subjects; nevertheless, these findings highlight the potential of a novel capsaicin-loaded nanoemulgel formulation for transdermal delivery in the management of the diabetic neuropathy.

**Figure 14:**
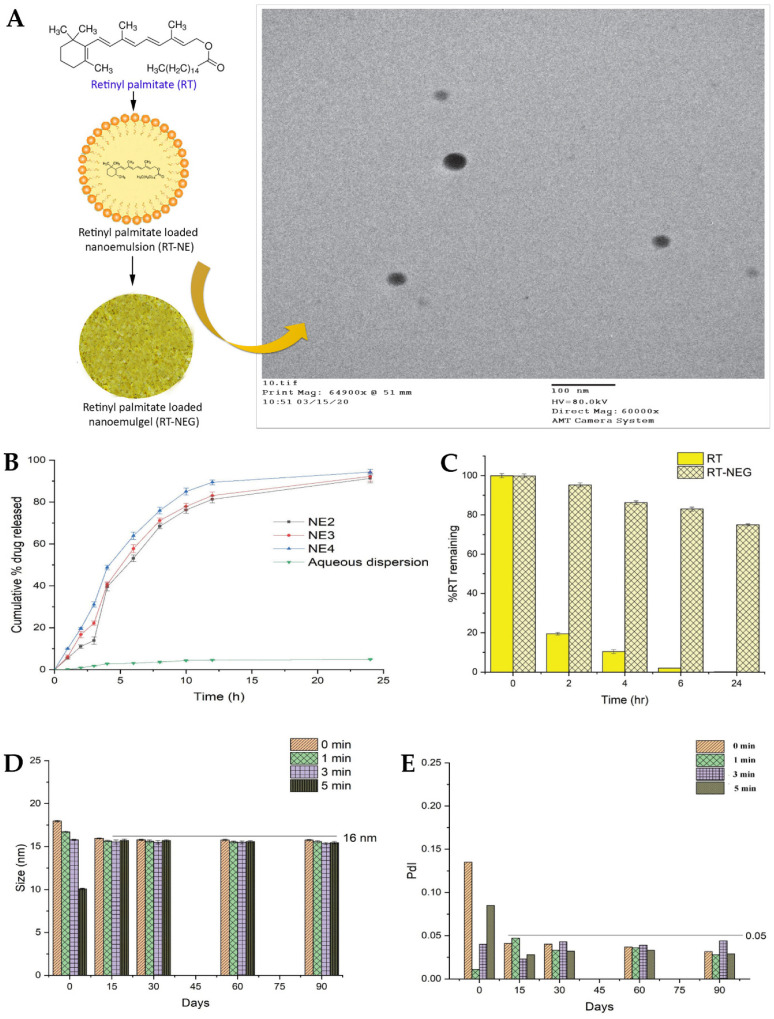
(**A**) Schematic representation of the developed retinyl palmitate topical nanoemulgel (left), with corresponding transmission electron microscopy image showing droplet morphology and size (right); (**B**) in vitro drug release profiles of different retinyl palmitate-loaded preliminary nanoemulsions, compared to an aqueous dispersion of the drug; (**C**) UV stability of the developed retinyl palmitate nanoemulgel, compared to the nonencapsulated drug; (**D**) mean droplet size variations of the preliminary optimized retinyl palmitate-loaded nanoemulsions as effect of storage time; (**E**) PDI variations of the preliminary optimized retinyl palmitate-loaded nanoemulsions as effect of storage time; NE—nanoemulsion; NEG—nanoemulgel; PDI—polydispersity index; RT—retinyl palmitate; adapted from Algahtani et al. [163], reproduced with permission from MDPI (Creative Commons CC BY 4.0 470 license).

Retinyl palmitate (RT), a derivate of vitamin A, has shown potential for skin rejuvenation and the treatment of various dermatological conditions, such as acne, psoriasis, ichthyosis, wrinkles, dark spots, and skin aging [251,252,253]. This molecule has the capability of exfoliating the surface layer of the skin and speeding up cell turnover, increasing skin moisture, and decreasing skin wrinkles, resulting in the skin looking fresher, smoother, and younger, and also acting as an antioxidant when applied topically, preventing tissue atrophy, and having anti-inflammatory effects [254,255,256]. Nevertheless, in addition to its good properties, RT also has side effects, such as skin irritation, redness, excessive peeling and dryness, being toxic in higher concentrations, while also being a lipophilic compound, with poor aqueous solubility, and limited skin permeation, which all hinder its therapeutic efficacy [257,258,259]. To overcome these limitations, Algahtani et al. [163] focused on the development and evaluation of a nanoemulgel formulation for enhanced topical delivery of RT (Figure 14A), to improve its solubility, stability, and permeation for antiaging effects. First, preliminary RT-loaded nanoemulsions were prepared, using a low-energy emulsification technique, by mixing an optimized oil and surfactant mixture phase with an aqueous phase, using a vortex mixer. RT showed good solubilization in different oils, surfactants, and cosurfactants, which were then selected based on their good miscibility with each other, but also on the resulting hydrophile–lipophile balance value. This was found to be optimum with an oil phase combining medium-chain monoglycerides (Capryol^®^ 90) and medium-chain triglycerides (Captex^®^ 355) in a 2:1 ratio, and a surfactant/cosurfactant mixture made of Kolliphor^®^ EL and Transcutol^®^ HP. The oil concentration affected the mean droplet size of the produced nanoemulsions, which increased with increasing oil concentration from 10% to 20%. Oil percentage also affected the PDI value, which also increased when increasing the oil phase percentage. On the other hand, variations in oil and surfactant/cosurfactant concentrations did not significantly affect zeta potential values. Viscosity values ranged from 77 to 89 cP, which showed that the viscosity remained constant and the nanoemulsions behaved as fluids. The selected preliminary formulations were further evaluated in in vitro drug diffusion studies (Figure 14B) using the dialysis bag method, with aliquots being collected at different time intervals for 24 h and analyzed by UV-spectroscopy. The drug release from the nanoemulsion systems was high and almost complete, ranging from 89% to 94%, while the release from the aqueous drug dispersion ended up being around 10 times smaller. Nanoemulsion storage stability was also tested, with droplet size and PDI values being monitored. Formulations remained stable for a period of 90 days, with the measured parameters remaining relatively constant (Figure 14D,E). The selected optimized nanoemulsion had a droplet size of 16.71 nm (Figure 14A), a PDI value of 0.015, and a zeta potential of −20.6 mV, with a measured drug percentage of approximately 99.0% and the highest cumulative drug release, and was selected for converting into a nanoemulgel system. The nanoemulgel formulation was prepared by incorporating the optimized nanoemulsion into a gel matrix, using Carbopol^®^ 940 (0.5% *w*/*w*) as the gelling agent, and with the addition of glycerin as humectant and to provide a smooth and soothing sensation upon topical application. The pH of the nanoemulgel was 5.53 ± 0.06, which is similar to the skin’s pH and is optimal for not causing skin irritation. The developed nanoemulgel exhibited non-Newtonian, pseudoplastic behavior, shear thinning, and thixotropic properties, which are convenient characteristics for topical application. Good spreadability is also important in achieving straightforward application of topical formulations, and results showed good spreadability for the developed nanoemulgel, with spreadability increasing with a higher applied force. Additionally, a stability evaluation of the developed nanoemulgel was performed, under different storage conditions, including temperature variations and exposure to light. The formulation exhibited excellent physical stability, with no significant changes observed in visual aspect, particle size, viscosity, or pH over the study period, indicating its robustness and long-term stability. Moreover, UV stability studies were done by measuring the percentage of RT remaining after an exposure to UV-A radiation (Figure 14C). A significant decrease in the amount of RT was evident after only 2 h, with the quantified amount reducing down to 19.46% in the control formulation, with no encapsulation of the drug. In contrast, 95.24% of the RT remained in the nanoemulgel formulation after 2 h of exposure, and 82.96% after 6 h, hence showing the potential of the developed formulation to encapsulate the drug and successfully protect it from photodegradation. Furthermore, the Franz diffusion cells were used again, this time to evaluate the ex vivo drug permeation of the developed nanoemulgel through excised dorsal rat skin. The nanoemulgel formulation demonstrated significantly enhanced skin permeation when compared to a conventional gel formation, indicating improved drug delivery and potentially enhanced therapeutic efficacy, with higher permeation coefficient.

## 3. Novel Nanoemulgels for Skin Application: The Future of Topical and Transdermal Drug Delivery?

Molecules that have been recently incorporated into nanoemulgels for skin application include repurposed marketed drugs, such as atorvastatin for wound healing [149], omeprazole for skin infections [155], and leflunomide for skin inflammatory diseases [146]. Natural-derived compounds have also had increased attention, with eucalyptol [150], naringenin [151], thymoquinone [152], and curcumin [153] being formulated for wound healing purposes, curcumin [153] and chrysin [142] for skin cancer, brucine [161] for skin inflammatory diseases, and capsaicin for neuropathy [162]. Other encapsulated compounds include marketed drugs such as miconazole [154], ketoconazole [157], and fusidic acid [158] for the treatment of skin and skin appendage infections, imiquimod [159] for skin cancer, and meloxicam [160] for skin inflammatory diseases, as well as the synthetic compound ebselen [156] as an anti-infection agent, vitamin E derivative tocotrienols for wound healing [151], and retinyl palmitate [163] for antiaging purposes. In what concerns formulation aspects, although spontaneous emulsification was sometimes used in the analyzed studies, most had to resort to high-pressure or high-speed homogenization, sometimes followed by ultrasonication, probably due to the formulations’ components not having ideal miscibility and molecular compatibility, and hence not being able to form nanoemulsions without the help of high-energy methods. In what concerns formulation components, various oils were used, with eucalyptus oil, black seed oil, and myrrh oil being the most applied, followed by other natural-derived oils, such as almond oil or olive oil, as well as oleic acid, liquid paraffin, and Captex 300 EP/NF or Captex 355. The oils were either selected based on potential biocompatibility, highest drug/active compound solubilization capacity, or both. Hydrophobic surfactants were also at times used as oils, replacing the latter, with Capryol 90 being the most used, followed by Span 80 or Span 60, and Labrafac PG or Labrafac Lipophile WL1349. The advantages of using hydrophobic surfactants with the additional function of oils reside in the fact that generally a highest drug solubilization is achieved, hence increasing the obtainable final formulation drug strength, and also in the fact that the absence of a hydrophobic component with no surfactant capability increases the chances of obtaining a nanoemulsion/nanoemulgel with lower droplet size and PDI, also augmenting the chances for spontaneous emulsification to be possible (instead of having to resort to high-energy fabrication methods). In the hydrophilic surfactants category, Tween 80 was by far the most applied, followed by Kolliphor EL, with Tween 20 and Solutol HS15 also being used. All these surfactants have been proven to have the capacity to increase skin drug permeation and retention by potentially being able to increase the skin barrier lipids’ fluidity and hence reduce the *stratum corneum*’s barrier function, hence making them adequate for skin drug delivery [260,261,262,263]. In what concerns cosolvents/cosurfactants, Transcutol was the most utilized. This potent cosolvent has also been reported to readily penetrate the *stratum corneum*, and once there, to modify its molecular mobility, namely, the protein and lipids that are part of its composition, hence decreasing the skin’s barrier function [264,265]. Other cosolvents/cosurfactants included PEG 400, propylene glycol, ethyl alcohol, isopropyl alcohol, Soluplus, and dimethylacetamide. And, finally, in what concerns gelling agents, the most used were in fact Carbopol varieties, such as Carbopol 940, Carbopol 934, or Carbopol Ultrez 21, followed by cellulose derivatives, such as CMC or HPMC, and with Pluronic F127 and chitosan also being applied. These gelling agents increase the formulations’ viscosity, hence resulting in an adequate consistency for topical application, with some also having bioadhesive properties, hence increasing the time of formulation retention at the application site, and with others even having drug permeation enhancement capacity as well, therefore contributing to a potentially better therapeutic outcome [266,267,268].

Regardless of formulation composition or preparation method, all developed nanoemulgels exhibited small droplet size and PDI values, resulting in good stability during storage or under stress conditions, adequate viscosity and spreadability, pH values compatible with the skin, controlled drug release profiles, and adequate ex vivo skin drug permeation and/or retention, at times even performing better than currently marketed formulations. Furthermore, in vitro and/or in vivo studies confirmed the safety and efficacy of the developed formulations for anti-infectious, anti-inflammatory, antitumor, wound-healing, antinociceptive, and/or antiaging effects, confirming their efficacy and therapeutic potential, and making them promising platforms for the replacement of current therapies, or as possible adjuvant treatments.

While clinical application is still further down the road, these systems have proven to have great potential for skin drug delivery. More work should be done to assess these formulations’ true safety and long-term applicability, in the context of the current regulatory framework, in order to determine the ability of these nanoformulations for bioaccumulation and interference with the body’s immune system (phagocytosis by immune cells), although this might be a bigger concern for transdermal drug delivery than for topical drug delivery [269,270]. Additionally, scale-up potential should also be determined, in order to assess the limitations facing any transition from a laboratory bench scale to an industrial manufacturing scale, so that these promising preparations can one day reach the pharmaceutical market [108,271,272,273].

## 4. Conclusions

Novel topical and transdermal nanoemulgels have been developed for skin application, encapsulating a wide variety of molecules, such as already marketed drugs (miconazole, ketoconazole, fusidic acid, imiquimod, meloxicam), repurposed marketed drugs (atorvastatin, omeprazole, leflunomide), natural-derived compounds (eucalyptol, naringenin, thymoquinone, curcumin, chrysin, brucine, capsaicin), and other synthetic molecules (ebselen, tocotrienols, retinyl palmitate), for wound healing, skin and skin appendage infections, skin inflammatory diseases, skin cancer, neuropathy, or antiaging purposes. All developed nanoemulgels had adequate droplet size, PDI, viscosity, spreadability, pH, stability, drug release, and drug permeation and/or retention capacity, having more advantageous characteristics than currently marketed formulations. In vitro and/or in vivo studies confirmed the safety and efficacy of the developed formulations, confirming their therapeutic potential and making them promising platforms for the replacement of current therapies, or as possible adjuvant treatments. Further studies will tell if these novel functional platforms for drug delivery might someday effectively reach the market to help fight highly incident skin or systemic diseases and conditions.

## Figures and Tables

**Table 1:** Summary of the most relevant analyzed information of each included study, namely, disease intended to treat, encapsulated molecule(s), main formulation composition, droplet size, PDI, ZP, pH, main in vitro and/or in vivo therapeutic efficacy-related results, and corresponding reference.

Disease Intended to Treat	Encapsulated Molecule(s)	Main Formulation Composition	Droplet Size (nm)	PDI	ZP (mV)	pH	Main In Vitro and/or In Vivo Therapeutic Efficacy-Related Results	Reference
Skin cancer	Chrysin	Capryol^®^ 90, Tween^®^ 80, Transcutol^®^ HP, Pluronic^®^ F127, water	<300	0.26	−15	NR	Strong antiproliferative effect in human and murine melanoma and human epidermoid carcinoma cell lines	[142]
Skin cancer and psoriasis	Leflunomide	Capryol^®^ 90, Cremophor^®^ EL, Transcutol^®^ HP, Pluronic^®^ F-127, water	98.7 to 280.92	0.2 to 0.3	−7.8	NR	Significant antimelanoma activity by inducing apoptosis and inhibiting tumor cell proliferation in melanoma cells; potent antipsoriatic activity by inhibiting human keratinocyte proliferation and reducing proinflammatory cytokine levels	[146]
Skin wound healing	Atorvastatin	Liquid paraffin, Tween^®^ 80, propylene glycol, carboxymethyl cellulose, water	100 to 200	<0.300	−20 to −30	7.6 to 7.8	Positive effect on wound healing, showing reduced inflammation, increased angiogenesis, and marked improvement in the skin’s histological architecture, after topical application on rat skin for 21 days	[149]
Skin wound healing	Eucalyptus oil	Black seed oil, Tween^®^ 80, Span^®^ 60, propylene glycol, Carbopol^®^ 940, water	139 ± 5.8	<0.450	−28.05	5 to 6	Significant improvement in wound healing in a rabbit model, with almost complete wound contraction after a 15-day period	[150]
Skin wound healing	Tocotrienols and naringenin	Capryol^®^ 90, Solutol^®^ HS15, Transcutol^®^ P, Carbopol^®^ 934 or Carbopol^®^ 940, water	145.6 ± 12.5	0.452 ± 0.03	−21.1 ± 3.32	4.9 to 5.3	NR	[151]
Skin wound healing	Thymoquinone	Black seed oil, Kolliphor^®^ EL, Transcutol^®^ HP, Carbopol^®^ 940, water	40.02 to 99.66	0.052 to 0.542	−26.7 to −30.6	5.53 ± 0.04	Accelerated wound closure in an in vivo rat wound model, evidenced by reduced wound size, enhanced re-epithelization, and increased collagen deposition	[152]
Skin wound healing	Curcumin	Labrafac™ PG, Tween^®^ 80, PEG 400, Carbopol^®^ 940, water	49.61 to 84.23	0.10 to 0.23	−15.96 ± 0.55 to −20.26 ± 0.65	5.53 ± 0.03	Significant wound-healing activity in Wistar rats, with almost complete wound healing after 20 days, with reduced inflammatory cells and extensive collagen fiber production	[153]
Skin infections	Miconazole nitrate	Olive oil, almond oil, Tween^®^ 80, Span^®^ 80, Carbopol^®^ 940, water	170	0.193	<−30	NR	Significant antifungal activity against selected fungal strains (*Candida albicans*)	[154]
Skin infections	Omeprazole	Olive oil, Span^®^ 80, Tween^®^ 80, chitosan, water	369.7 ± 8.77	0.316	−15.3 ± 6.7	6.21 ± 0.21	Substantial antibacterial effects against both Gram-negative bacteria (*Escherichia coli*, *Klebsiella pneumoniae*, *Pseudomonas aeruginosa*) and Gram-positive bacteria (*Staphylococcus aureus*)	[155]
Skin infections	Ebselen	Captex^®^ 300 EP/NF, Kolliphor^®^ ELP, dimethylacetamide, Soluplus^®^, Aquaphor, water	54.82 ± 1.26	NR	−1.69	NR	Potent antifungal activity against multi-drug-resistant *Candida albicans* and *Candida tropicalis*	[156]
Skin appendage infections (nails)	Ketoconazole	Labrafac™ Lipophile WL1349, Polysorbate 80, PEG 400, Carbopol^®^ Ultrez 21, glycerin, methylparaben, thioglycolic acid, aminomethyl propanol, water	77.52 ± 0.92	0.128 ± 0.035	−5.44 ± 0.67	6.4 ± 0.24	Significant antifungal activity against clinical isolates of dermatophytes, namely, *Trichophyton rubrum* and *Candida albicans*	[157]
Skin infections	Fusidic acid	Myrrh oil, Tween^®^ 80, Transcutol^®^ P, CMC, water	116 to 226	NR	NR	6.61 ± 0.23	Substantial antibacterial activity, with significant inhibition zones against *Staphylococcus Aureus*, *Bacillus subtilis*, *Enterococcus faecalis*, *Candida albicans*, *Shigella*, and *Escherichia coli*	[158]
Skin cancer	Imiquimod and curcumin	Oleic acid, Tween^®^ 20, Transcutol^®^ HP, Carbopol^®^ 934, water	78.39	0.254	−18.7	5.5	Did not lead to the appearance of psoriasis-like symptoms after topical application to mice	[159]
Skin inflammatory diseases	Meloxicam	Eucalyptus oil, Tween^®^ 80, PEG 400, Transcutol^®^ P, distilled water	139 ± 2.31 to 257 ± 3.61	NR	NR	6.58 ± 0.21	Confirmed anti-inflammatory effects with reduced inflammation percentage in in vivo study	[160]
Skin inflammatory diseases	Brucine	Myrrh oil, Tween^®^ 80, PEG 400, ethyl alcohol, carboxymethyl cellulose, water	151 ± 12	0.243	NR	6.2 ± 0.2	Anti-inflammatory effects (significant decrease in inflammation) and antinociceptive effects (reduction in writhing movements) in an animal model, after topical application	[161]
Diabetic neuropathy	Capsaicin	Eucalyptus oil, Tween^®^ 80, propylene glycol, ethanol, isopropyl alcohol, Carbopol^®^ 940, water	28.15 ± 0.24	0.27 ± 0.05	NR	NR	Significant in vivo efficacy in alleviating mechanical allodynia	[162]
Skin aging	Retinyl palmitate	Capryol^®^ 90, Captex^®^ 355, Kolliphor^®^ EL, Transcutol^®^ HP, Carbopol^®^ 940, glycerin, water	16.71	0.015	−20.6	5.53 ± 0.06	NR	[163]

CMC—carboxymethyl cellulose; NR—not reported; PDI—polydispersity index; PEG—polyethylene glycol; ZP—zeta potential.

## Data Availability

Not applicable.

## Abbreviations

ATR—atorvastatin; CMC—carboxymethyl cellulose; CNTs—carbon nanotubes; CUR—curcumin; DMA—dimethylacetamide; EB—ebselen; FA—fusidic acid; GRAS—generally regarded as safe; HPMC—hydroxypropyl methylcellulose; IMQ—imiquimod; MIC—minimum inhibitory concentration; MSNs—mesoporous silica nanoparticles; MX—meloxicam; NPs—nanoparticles; O/W—oil-in-water; PBS—phosphate buffer saline; PCL—polycaprolactone; PDI—polydispersity index; PEG—polyethylene glycol; PLGA—poly(lactic-co-glycolic acid; PMs—polymeric micelles; PNPs—polymeric nanoparticles; RT—retinyl palmitate; SEM—scanning electron microscopy; SLNs—solid lipid nanoparticles; TMQ—thymoquinone; W/O—water-in-oil; UV—ultraviolet.
